# AfLaeA, a Global Regulator of Mycelial Growth, Chlamydospore Production, Pathogenicity, Secondary Metabolism, and Energy Metabolism in the Nematode-Trapping Fungus *Arthrobotrys flagrans*

**DOI:** 10.1128/spectrum.00186-23

**Published:** 2023-06-26

**Authors:** Yu Zhang, Xin Wang, Yuan Ran, Ke-Qin Zhang, Guo-Hong Li

**Affiliations:** a State Key Laboratory for Conservation and Utilization of Bio-Resources in Yunnan, Key Laboratory for Microbial Resources of the Ministry of Education, School of Life Sciences, Yunnan University, Kunming, China; University of Wisconsin-Madison

**Keywords:** nematode-trapping fungi, *Arthrobotrys flagrans*, genome, AfLaeA, chlamydospore, pathogenicity, metabolism

## Abstract

Arthrobotrys flagrans (*Duddingtonia flagrans*) is a typical nematode-trapping fungus which has been used for nematode biocontrol. The global regulator LaeA is widely distributed in filamentous fungi and plays a crucial role in secondary metabolism and development in addition to pathogenicity in fungal pathogens. In this study, the chromosome-level genome of *A. flagrans* CBS 565.50 was sequenced and homologous sequences of LaeA were identified in *A. flagrans*. *A. flagrans LaeA* (*AfLaeA*) knockout resulted in slower hyphal growth and a smoother hyphal surface. Importantly, deletion of *AfLaeA* resulted in the absence of chlamydospores and attenuated glycogen and lipid accumulation in hyphae. Similarly, disruption of the *AfLaeA* gene led to fewer traps and electron-dense bodies, lower protease activity, and a delay in capturing nematodes. The *AfLaeA* gene had a large effect on the secondary metabolism of *A. flagrans*, and both the deletion and overexpression of *AfLaeA* could yield new compounds, whereas some compounds were lost due to the absence of the *AfLaeA*. Protein-protein interactions between AfLaeA and another eight proteins were detected. Furthermore, transcriptome data analysis showed that 17.77% and 35.51% of the genes were influenced by the *AfLaeA* gene on days 3 and 7, respectively. *AfLaeA* gene deletion resulted in the higher expression level of the *artA* gene cluster, and multiple differentially expressed genes involved in glycogen and lipid synthesis and metabolism showed opposite expression patterns in wild-type and Δ*AfLaeA* strains. In summary, our results provide novel insights into the functions of *AfLaeA* in mycelial growth, chlamydospore production, pathogenicity, secondary metabolism, and energy metabolism in *A. flagrans*.

**IMPORTANCE** The regulation of biological functions, such as the secondary metabolism, development, and pathogenicity of LaeA, has been reported in multiple fungi. But to date, no study on LaeA in nematode-trapping fungi has been reported. Moreover, it has not been investigated whether or not LaeA is involved in energy metabolism and chlamydospore formation has not been investigated. Especially in the formation mechanism of chlamydospores, several transcription factors and signaling pathways are involved in the production of chlamydospores, but the mechanism of chlamydospore formation from an epigenetic perspective has not been revealed. Concurrently, an understanding of protein-protein interactions will provide a broader perspective on the regulatory mechanism of AfLaeA in *A. flagrans*. This finding is critical for understanding the regulatory role of AfLaeA in the biocontrol fungus *A. flagrans* and establishes a foundation for developing high-efficiency nematode biocontrol agents.

## INTRODUCTION

The growth, secondary metabolism, and sexual and asexual development of filamentous fungi are regulated by genetic regulators (1). LaeA (loss of *aflR* expression A) is a global regulator genetically identified from Aspergillus nidulans (2) and is now known to be a phylogenetically conserved methyltransferase in multiple filamentous fungi species (3). Generally, an *S*-adenosylmethionine (SAM) binding site at the N terminus and a methyltransferase domain at the middle of the LaeA protein are present. Moreover, LaeA protein has a classical nuclear localization signal, which localizes into the nucleus (2). In A. nidulans, LaeA regulates secondary metabolism and fungal development by forming a trimeric protein complex with VeA and VelB in response to light (1). Similarly, Penicillium oxalicum LaeA (PoLaeA) forms a complex with three proteins (Tup1, Cyc8, and PoClrB) to modify chromatin structure in the upstream region of cellulose degradation genes, thereby activating the expression of the cellulose degradation gene in *Penicillium oxalicum* (3). In more detail, LaeA regulates the expression of *cexA* via methylation levels of the histones H3K4 and H3K9 in Aspergillus luchuensis (4).

Initially, most of the research on LaeA has been performed in Aspergillus spp., where LaeA is involved in the production of multiple secondary metabolites such as sterigmatocystin, penicillin, and lovastatin (2). Subsequent work revealed that LaeA regulates the production of secondary metabolites of *Penicillium* spp. (5, 6), *Trichoderma* spp. (7, 8), the endophytic fungus Epichloe festucae (9), and multiple pathogenic fungi, such as Dothistroma septosporum (10), Alternaria alternata (11), Fusarium oxysporum (12), Fusarium verticillioides (13), and Magnaporthe oryzae (14). The secondary metabolites of pathogenic fungi are closely related to their pathogenicity, and the pathogenicity of multiple pathogenic fungi is also positively regulated by LaeA. For example, deletion of the *VmLaeA* gene was found to lead to a significant reduction in the virulence of Valsa mali for tobacco (15). Similarly, loss of the LaeA gene in A. alternata (16) and Cochliobolus heterostrophus (17) resulted in a reduced ability to infect tomato and maize leaves, respectively. In addition, the *PeLaeA* gene mutant strain from *Penicillium expansum* failed to colonize on apples, and patulin was not detected in apples infected with the mutant strain (18).

LaeA not only regulates the synthesis of fungal secondary metabolites and virulence but also has key functions in fungal development, including the formation of asexual spore and sexual fruiting bodies (1, 14). In A. nidulans, the absence of *AnLaeA* results in the lack of Hülle cells, a specific globose cell type, which nurse the young fruiting body during development. Because of the absence of this gene, fruiting bodies formed in AnLaeA mutant strains are smaller (19, 20). Similarly, loss of *MrLaeA* results in the lack of ascospores in Monascus ruber (21). In addition, LaeA is essential to support asexual spore formation in A. nidulans (19, 20), Aspergillus pachycristatus (22), and Aspergillus carbonarius (23), and the number of asexual spores in LaeA mutant strains showed a significant decrease in the light. Apart from Aspergillus spp., LaeA is evidently essential for sporulation in other filamentous fungi. LaeA positively regulates the conidial production of *Trichoderma* spp., including Trichoderma longibrachiatum SMF2Shi, T. atroviride, and T. reesei (24, 25), *Penicillium* spp., including *P. oxalicum*, P. chrysogenum, and P. expansum (26–28), Chaetomium globosum (29), A. alternata (16), and Ganoderma lingzhi (30) but negatively regulates the conidial production of *C. heterostrophus* (17) and *M. ruber* (21). LaeA is crucial for development in filamentous fungi.

As a major source for the development of biological nematicides, nematode-trapping (NT) fungi capture nematodes by forming specialized trapping devices (traps), such as adhesive networks, knobs, and constricting rings (31). *Arthrobotrys flagrans* (formerly *Duddingtonia flagrans*) is a typical NT fungus that is easy to cultivate and has a strong capability to capture nematodes by producing adhesive networks (31, 32). Importantly, the capability of *A. flagrans* to produce a large number of chlamydospores is a unique advantage over other NT fungi (33). Biocontrol agents developed from this strain have been successfully used to control parasitic nematodes in multiple animals such as cattle, sheep, horses, chickens, and pigs, among others (34). It is believed that *A. flagrans* has the potential for development as a biocontrol agent for plant-parasitic nematodes, such as *Meloidogyne* spp. and Xiphinema index (32–35). LaeA is widespread in filamentous fungi (14, 36), whereas its function remains unclear in NT fungi.

In this study, based on chromosome-level genome sequencing, we identified *AfLaeA* in the NT fungus *A. flagrans*. The effects of AfLaeA on hyphal growth, chlamydospore production, virulence, and secondary and energy metabolic processes were characterized by gene knockout, gene complementation, and multiphenotype and transcriptome data analysis.

## RESULTS

### Chromosome-level genome assembly and annotation of *A. flagrans*.

**FIG 1:**
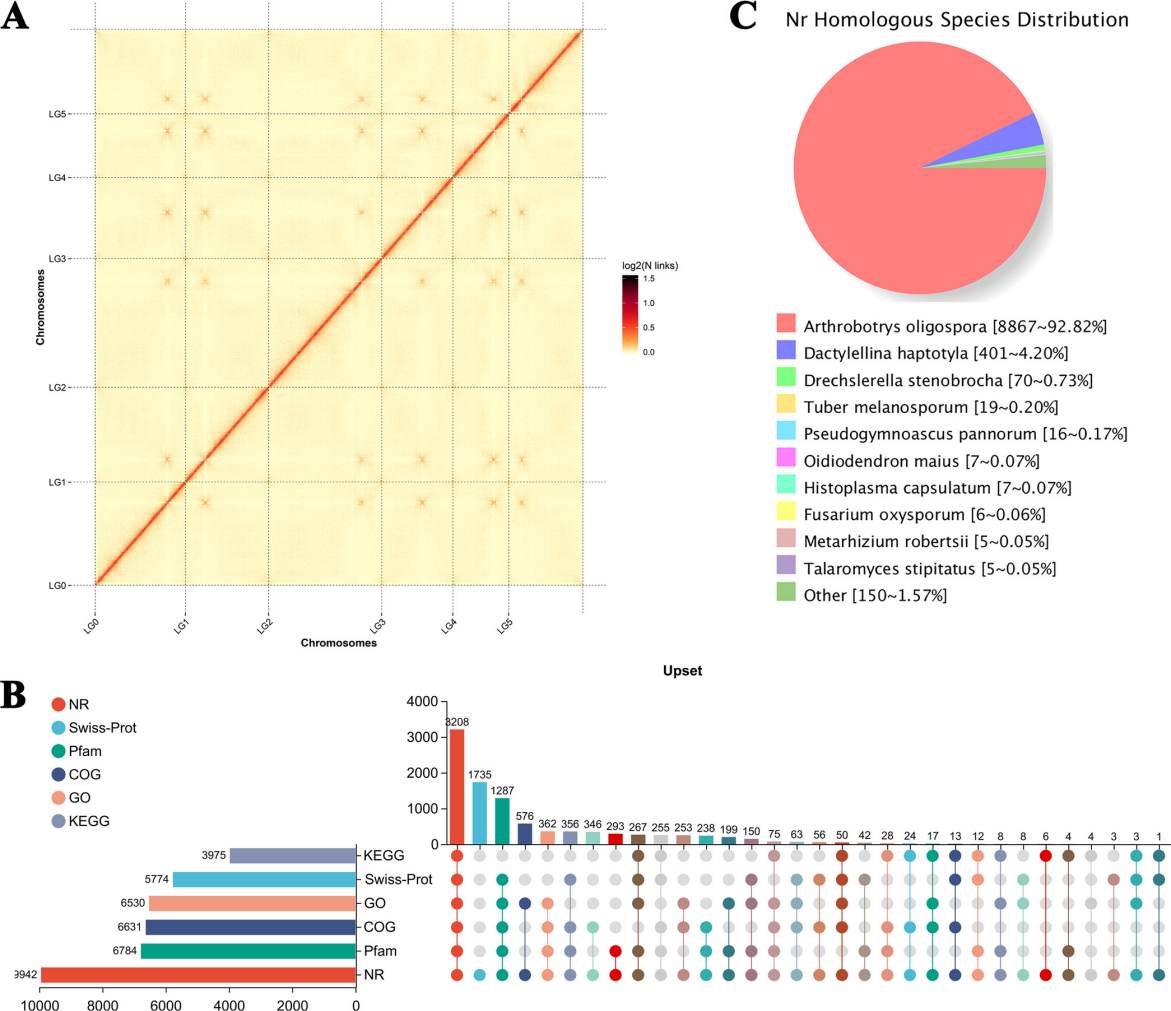
Chromosome-level genome assembly and annotation of *Arthrobotrys flagrans*. (A) Interactive heat map of Hi-C assembled chromosomes. LG00 to LG05 represent lachesis groups 00 to 05, and the abscissa and ordinate represent the order of each bin on the corresponding chromosome group. (B) The UpSet diagram of gene annotation statistics. The horizontal histogram on the left shows the statistical values of elements of each set. In the middle matrix, single points represent the unique elements of a set, the connections between points represent the unique intersections of different sets, and the vertical histogram represents the corresponding intersection element values, respectively. (C) Species distribution map of protein sequences compared with the Nonredundant Protein (NR) database.

The first genome data (scaffold level) of *A. flagrans* CBS 349.94 were reported in 2019 (32). In the present study, the chromosome-level genome of strain *A. flagrans* CBS 565.50 was sequenced and analyzed. This study yielded 8.91 Gb of initially filtered PacBio data, and 99.07% of reads were mapped against the genome of *A. flagrans* CBS 349.94 with 109.58× genome coverage (Table 1). The genome sequence of 35.85 Mb was then assembled using Hifiasm software, and the genome was found to be located on six chromosomes (Fig. 1A), containing six lachesis groups with an *N*_50_ scaffold size of 6,108,558 bp and an *N*_90_ scaffold size of 4,110,191 bp (Table 1). Importantly, compared with the genome data of *A. flagrans* CBS 349.94, the gene cluster was found to have more protein-coding genes (up to 9,997) with an average gene size of 1,701.43 bp than found in the *A. flagrans* CBS 565.50 genome (Table 1). One hundred thirty-three effector proteins and 1,805 transmembrane proteins were also predicted. In addition, the *A. flagrans* CBS 565.50 genome has 11 secondary metabolism gene clusters, including four polyketide synthase (PKS), two nonribosomal peptide synthase (NRPS), and two terpene gene clusters and one siderophore, one indole, and one betalactone gene cluster (see Fig. S1 and Table S1 in the supplemental material), whereas the genome of *A. flagrans* CBS 349.94 has only three PKS and three predicted NRPS type gene clusters (32). Furthermore, 733 secreted proteins (secretome) were predicted in the *A. flagrans* CBS 565.50 genome, whereas 638 secreted proteins were predicted in the *A. flagrans* CBS 349.94 genome (32). Among these secreted proteins, small secreted proteins (SSPs) have been implicated in fungal pathogenicity (32), and the small secreted cysteine-rich protein CyrA was confirmed as a virulence factor participating in the attack of Caenorhabditis elegans in *A. flagrans* (37).

**TABLE 1:** Features of the chromosome-level genome of *A. flagrans* CBS 565.50

Sequencing feature	Value^*a*^
Genome size (Mb)	35.85
Chromosomes	6
Clean data (Gb)	8.91
Depth (×)	109.58
Mapped (%)	99.07
Coverage (%)	99.96
Lachesis groups	7
*N*_50_ read length (bp)	6,108,558
Max contig length (bp)	8,301,709
*N*_90_ length of scaffolds (bp)	4,110,191
% ≥ Q30	92.84
%G+C	45.57
Repeat content (%)	5.16
tRNA genes	177
rRNA genes	98
Total length of coding sequences (bp)	17,009,220
Avg gene size (bp)	1,701.43
Avg no. of exons/gene	3.61
Avg no. of introns/gene	2.61
Avg CDS size (codons)	403.46
Avg intron length (bp)	94.06
Protein-encoding genes	9,997
Gene clusters	11
No. (%) of secretomes	733 (~7.33)
Transmembrane proteins	1,805
Effector proteins	133

a Values are the numbers of the indicated feature unless otherwise specified.

To further understand gene function in *A. flagrans*, 6,530, 3,979, 6,631, 9,942, 5,774, and 6,784 proteins were successfully assigned to their orthologs in the Gene Ontology (GO), Kyoto Encyclopedia of Genes and Genomes (KEGG), Clusters of Orthologous Genes (COG), Nonredundant Proteins (NR), Swiss-Prot, and Pfam databases, respectively (Fig. 1B). There were 3,208 proteins that could be annotated in the six databases, and only 1,735 genes were found to belong to the NR database (Fig. 1B). Likewise, according to the comparison results of the NR database, *A. flagrans* and *Arthrobotrys oligospora* have the highest similarity, with 8,867 homologous sequences, and *A. flagrans* has 401, 70, 19, 16, 7, 7, 6, 5, and 5 sequences homologous with Dactylellina haptotyla, Drechslerella stenobrocha, Tuber melanosporum, Pseudogymnoascus pannorum, Oidiodendron maius, Histoplasma capsulatum, F. oxysporum, Metarhizium robertsii, and Talaromyces stipitatus, respectively (Fig. 1C).

### Identification and sequence analyses of AfLaeA and nine methyltransferases in *A. flagrans*.

**FIG 2:**
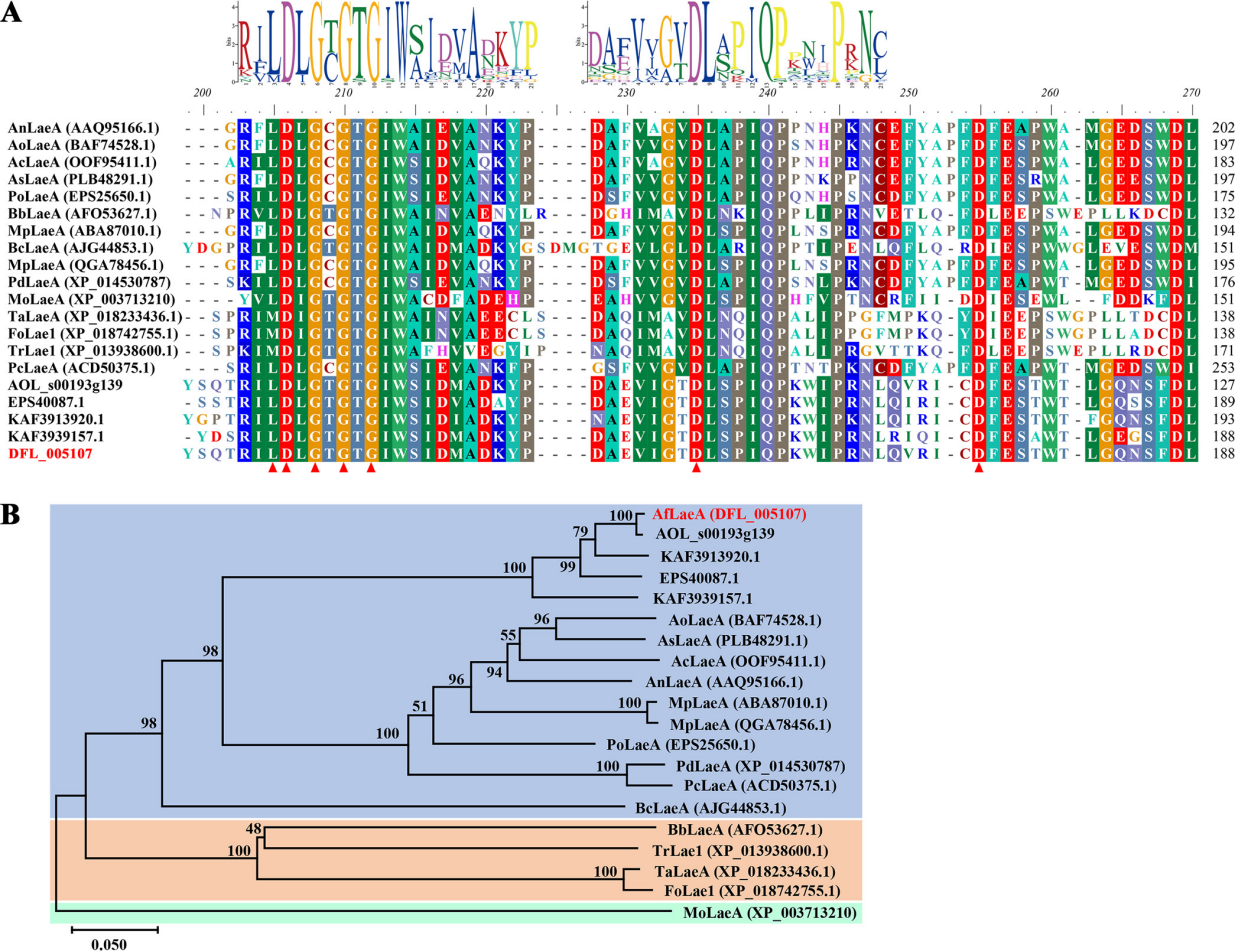
Conserved domains and phylogenetic analysis of LaeA in different fungi. (A) Homologous sequences of LaeA in different fungi were retrieved from the NCBI database, and the conserved domains were analyzed using BioEdit software. The GenBank accession numbers of the homologous sequences of LaeA in nematode-trapping fungi *A. oligospora*, *D. haptotyla*, *D. brochopaga*, and *D. cylindrospora* are XP_011127052.1, EPS40087.1, KAF3913920.1, and KAF3939157.1, respectively. The GenBank accession numbers of these LaeA proteins are shown in the figure. Red triangles indicate a conserved SAM binding site, and the logo of the conserved domain was predicted using MEME online software. (B) Phylogenetic tree constructed by MEGA X software using the neighbor-joining method. Bootstrap values based on 1,000 replicates are shown in the phylogenetic tree, and AfLaeA (DFL_005107) is highlighted in red.

Based on genomic data, the amino acid sequence of the A. nidulans global regulator AnLaeA (GenBank accession number AAQ95166.1) (1) was used for a BLAST search of the *A. flagrans* genome using National Center for Biotechnology and Information (NCBI) and UniProt software, and one AfLaeA protein (DFL_005107; GenBank accession number RVD86853.1) and nine methyltransferases were predicted, all of which contain a conserved SAM binding site (Fig. 2A; Table S2). AfLaeA and AnLaeA had the highest similarity, with 48.47% (Fig. 2A; Table S3), while the similarities of other methyltransferases were less than 37.92% (Table S3). The *AfLaeA* gene has seven exons and six introns, and the coding sequence (CDS) contains 1,518 bp that encodes 362 amino acids with a molecular weight of 41.491 kDa (Table S4). The subcellular localization prediction analysis revealed that AfLaeA and AnLaeA were localized to the cytoplasm and nucleus (Table S4). In addition, the homologous protein sequences of LaeA were also found in the nematode-trapping fungi *A. oligospora*, *D. haptotyla*, *D. brochopaga*, and *Dactylella cylindrospora* (Fig. 2A). They were clustered with AnLaeA in the phylogenetic tree (Fig. 2B). LaeA proteins from different species in the phylogenetic tree have been reported to be involved in biological processes such as fungal growth and development, conidial production, and secondary metabolism (Fig. 2).

### Deletion of *AfLaeA* affects fungal growth, morphology, and stress response in *A. flagrans*.

**FIG 3:**
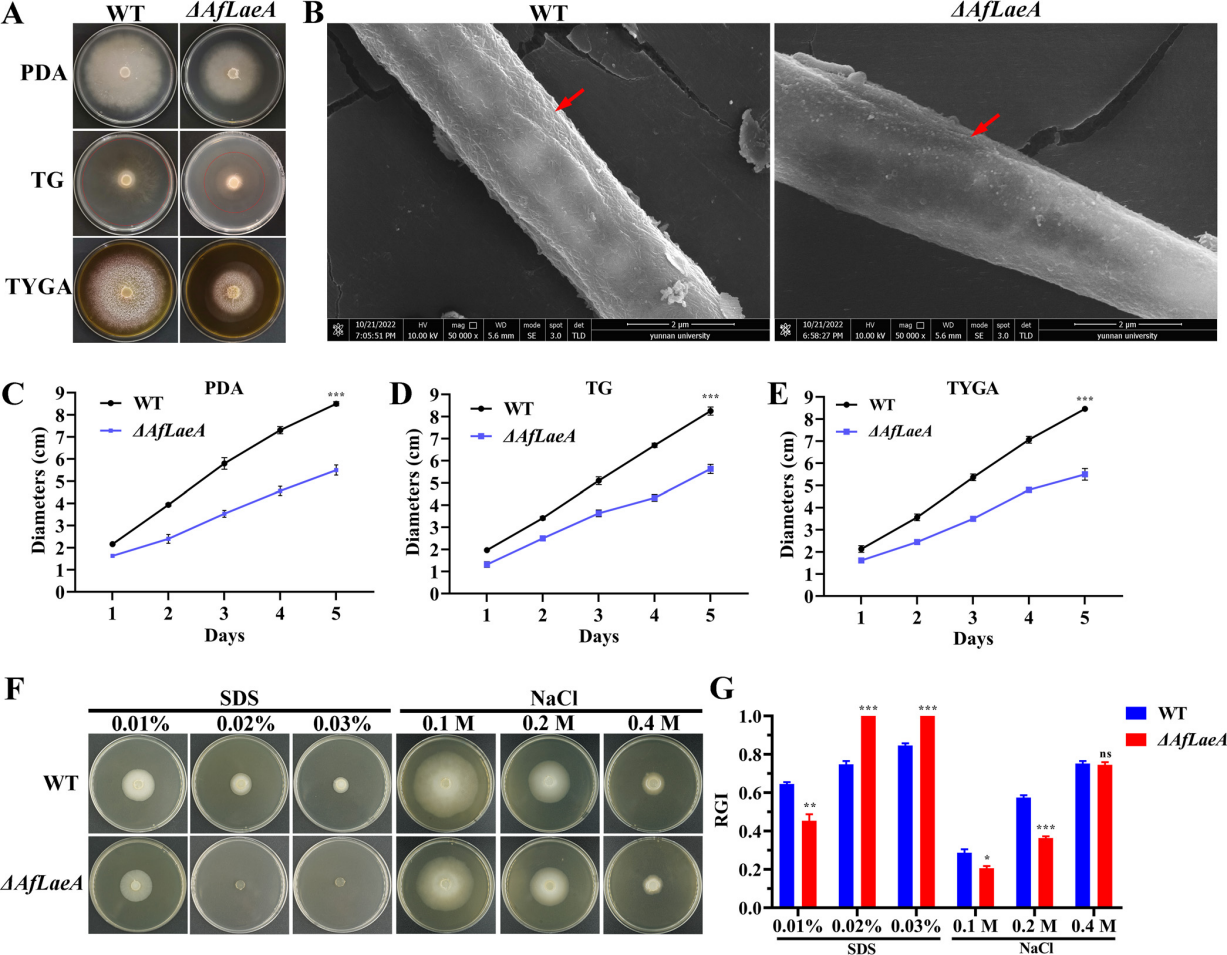
Comparison of growth, morphologies, and stress responses between WT and Δ*AfLaeA* strains. (A) Colony morphologies of the WT and Δ*AfLaeA* strains incubated on PDA, TG, and TYGA plates at 28°C for 5 days. (B) Analysis of the cell surface morphology of WT and Δ*AfLaeA* strains by SEM. Red arrows indicate wrinkled areas on the hyphal surface. (C to E) Colony diameters of the WT and Δ*AfLaeA* strains cultured on PDA, TG, and TYGA plates at 28°C for 5 days (***, *P *< 0.001). (F) Growth of the WT and Δ*AfLaeA* strains on medium supplemented with SDS at 0.01% to 0.03% and NaCl (0.1 M, 0.2 M, and 0.4 M). (G) RGI values of the WT and Δ*AfLaeA* strains on medium supplemented with SDS (0.01% to 0.03%) and NaCl (0.1, 0.2, and 0.4 M) (*, *P *< 0.05; **, *P *< 0.01; ***, *P *< 0.001).

In this study, we deleted the *AfLaeA* gene by a homologous recombination method to investigate its function in *A. flagrans* (Fig. S2). Likewise, three methyltransferase genes (*DFL_000451*, *DFL_006623*, and *DFL_007594*) were knocked out in the same way (Fig. S3). Deletion of *AfLaeA* gene resulted in significantly slower hyphal growth on potato dextrose agar (PDA), TG (1% tryptone, 1% glucose, 1.5% agar), and TYGA (1% tryptone, 0.5% yeast extract, 1% glucose, 0.5% molasses, 1.5% agar) media, with colony diameters of approximately 60% of that of the wild-type (WT) strain on day 5 (Fig. 3A and C to E). Complementation of *AfLaeA* restored colony growth (Fig. S5A and B). However, the loss of three methyltransferase genes (*DFL_000451*, *DFL_006623*, and *DFL_007594*) had no effect on hyphal growth (Fig. S4). Moreover, the *AfLaeA* gene was overexpressed in the WT strain, its expression was upregulated 3.5-fold (Fig. S5D and E), and AfLaeA was localized to both the nucleus and cytoplasm (Fig. S5F), which was consistent with the predicted results based on the Cell-PLoc software (Table S4). In addition, scanning electron microscopy (SEM) results showed that the surface of the Δ*AfLaeA* strain lacked wrinkles in comparison with the WT (Fig. 3B).

To confirm the effect of *AfLaeA* gene on stress resistance of *A. flagrans*, the osmotic pressure stress reagent NaCl (0.1, 0.2, and 0.3 M) and the cell wall stress reagent sodium dodecyl sulfate (SDS) at 0.01% to 0.03% were added to the PDA medium (Fig. 3F). The results showed that the growth of the WT and Δ*AfLaeA* strains could be significantly inhibited by 0.01% SDS and the growth of the Δ*AfLaeA* strain could be completely inhibited by 0.02% SDS (Fig. 3F). The Δ*AfLaeA* strain was more sensitive to SDS, with higher relative growth inhibition (RGI) values than those of the WT strain (Fig. 3F and G). Similarly, the growth of the WT and Δ*AfLaeA* strains could be inhibited by 0.1 M NaCl, and the WT strain displayed greater sensitivity to 0.1 M NaCl, with higher RGI values than those of the Δ*AfLaeA* strain (Fig. 3F and G). Interestingly, the WT and Δ*AfLaeA* strains had the same sensitivity to 0.4 M NaCl with the same RGI values (Fig. 3G).

### AfLaeA plays a key role in chlamydospore production in *A. flagrans*.

**FIG 4:**
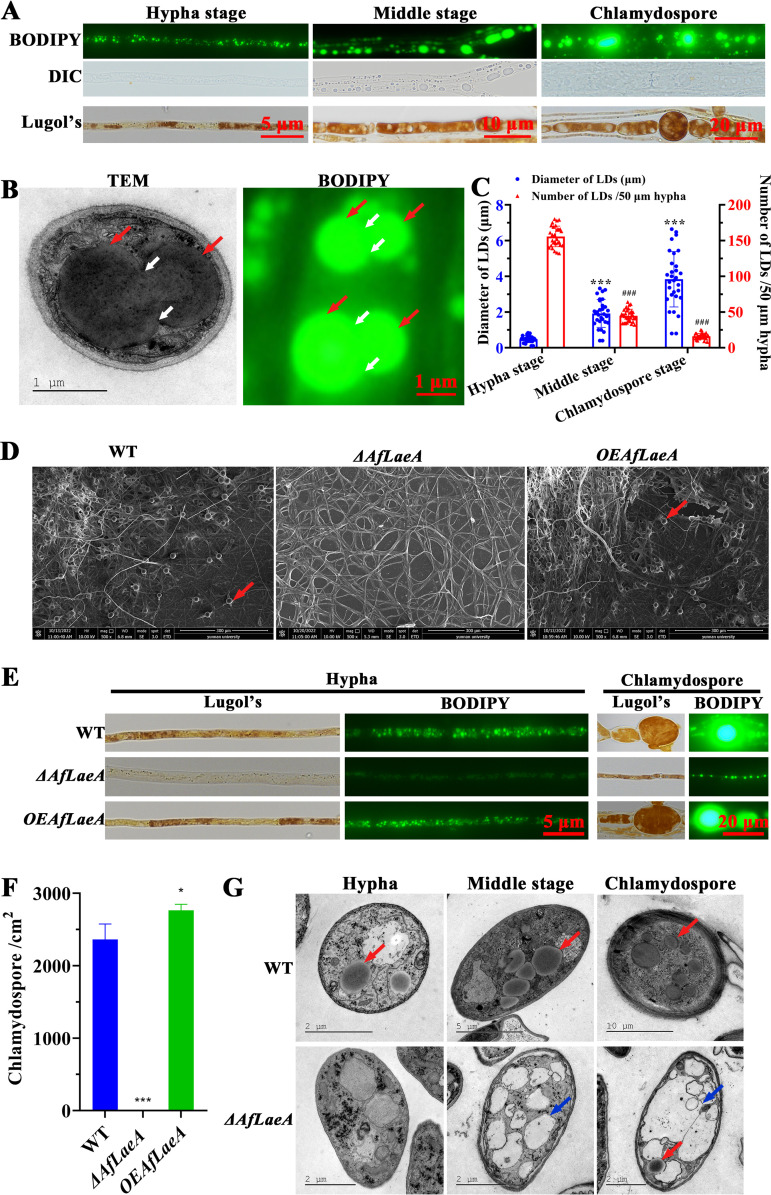
AfLaeA affects the formation of glycogen, LDs, and chlamydospores. (A) Staining analysis of glycogen and LDs at different stages (hyphal stage, middle stage of chlamydospore production, and chlamydospore stage) of chlamydospore formation. The LDs and glycogen were stained with BODIPY and Lugol’s iodine, respectively. DIC, differential interference contrast. (B) Observation of the fusion phenomenon of LDs by TEM and BODIPY staining. Red and white arrows indicate LDs and the gaps caused by the fusion of LDs, respectively. (C) Comparison of the diameters and numbers of the LDs in the process of chlamydospore formation. The diameters of LDs were collected from TEM images of more than 30 cells, and the number of LDs was obtained from a random hypha (50 μm) in more than 30 fields viewed under a microscope. The asterisks and pound signs indicate a significant difference between the hyphal stage and middle stage of chlamydospore production and the chlamydospore stage (Tukey’s honestly significant difference [HSD]; ***, *P *< 0.001; ###, *P *< 0.001). (D) Observation of the chlamydospores in WT, Δ*AfLaeA*, and *OEAfLaeA* strains using SEM. Red arrows indicate chlamydospores. (E) Comparison of the glycogen and LDs in WT, Δ*AfLaeA*, and *OEAfLaeA* strains in hyphal and chlamydospore stages, respectively. (F) Numbers of chlamydospores in WT, Δ*AfLaeA*, and *OEAfLaeA* strains (*, *P* < 0.05). (G) TEM analysis of the internal structure in WT and Δ*AfLaeA* strains at the hyphal and middle stages of chlamydospore production and the chlamydospore stage, respectively. Red and blue arrows indicate LDs and vacuoles, respectively.

In previous reports, LaeA was involved in the formation of conidia in several fungi (19–29), but it has not been reported to regulate chlamydospore formation. The chlamydospore is a prominent feature of *A. flagrans* strains. The strong stress resistance of chlamydospore has great significance for the biological control of nematodes. In this study, we observed the distribution of glycogen and lipid droplets (LDs) in hyphae and chlamydospores. In the hyphal stage, LDs in the cells were small and dispersed, but the volume was increased and the distribution was concentrated in the middle stage of chlamydospore production and in the chlamydospore stage (Fig. 4A). Importantly, the fusion phenomenon of LDs was observed by transmission electron microscopy (TEM) and BODIPY staining analysis (Fig. 4B). At the same time, the diameters of the LDs increased but the numbers of LDs decreased during formation of chlamydospores (Fig. 4C). This fully showed that the larger LDs were derived from the fusion of smaller LDs. Similarly, glycogen was also inappropriately increased during chlamydospore production, although we did not observe very significant fusion (Fig. 4A).

Importantly, deletion of *AfLaeA* gene resulted in the absence of chlamydospores, and this defect could be restored by complementation in the Δ*AfLaeA* strain (Fig. S5C). Interestingly, the overexpression (OE) of *AfLaeA* produced chlamydospores and increased their production by approximately 16% (Fig. 4D to F). In addition, the Δ*AfLaeA* strain showed defects in glycogen and LD formation and accumulation, although more glycogen and LDs were formed at the end of hyphal growth (Fig. 4E). Additionally, the accumulation of glycogen and LDs was not significantly increased in overexpressed *AfLaeA* (*OEAfLaeA*) strains (Fig. 4E). Of note, the deletion of a methyltransferase gene (*DFL_000451*) resulted in a 30% reduction in chlamydospores but had no significant effects on the accumulation of glycogen and LDs (Fig. S6). Furthermore, the accumulation of glycogen and LDs during chlamydospore formation in the WT strain was also observed using TEM, as shown in Fig. 4G. We also found that the loss of the *AfLaeA* gene resulted in the appearance of numerous vacuoles in cells at the same stage (Fig. 4G).

### Effect of AfLaeA on pathogenicity of *A. flagrans*.

**FIG 5:**
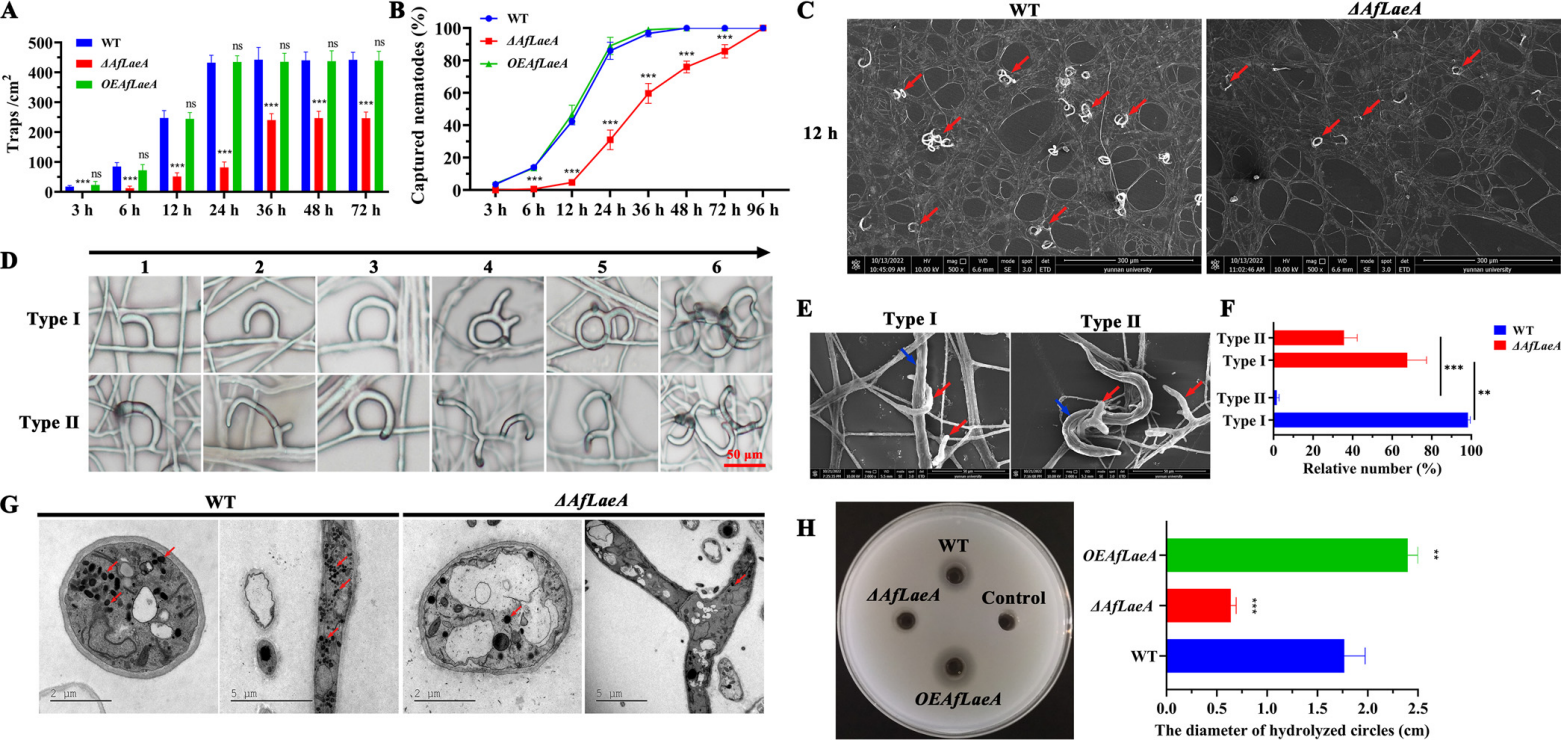
Comparison of trap formation, trap morphologies, nematocidal activities, EDs, and extracellular proteolytic activities. (A) Comparison of traps produced by WT, Δ*AfLaeA*, and *OEAfLaeA* strains at different time points (nonsignificant [ns], *P *> 0.05; *, *P *< 0.05; **, *P *< 0.01; ***, *P *< 0.001). (B) Percentage of captured nematodes at different time points. (C) SEM analysis of the traps at 12 h. Red arrows indicate traps. (D) Comparison of the trap morphologies in WT and Δ*AfLaeA* strains. The numbers at the top of the panel (1 to 6) represent the different stage of trap formation. (E) Nematodes were captured by two types of traps. Red and blue arrows indicate nematodes and traps, respectively. (F) Percentage of two types of traps in WT and Δ*AfLaeA* strains (***, *P *< 0.001). (G) Comparison of EDs in trap cells of WT and Δ*AfLaeA* strains based on TEM. Red arrows indicate EDs in trap cells of WT and Δ*AfLaeA* strains. (H) Comparison of extracellular protease activities (**, *P *< 0.01; ***, *P *< 0.001).

As an NT fungus, the pathogenicity of *A. flagrans* for nematodes is an important function (38, 39). In this study, the effects of the *AfLaeA* gene on the production of traps and the capability to capture nematodes were investigated. The results showed that deletion of *AfLaeA* gene resulted in significantly fewer traps and a lower production rate of traps (Fig. 5A and C). After the addition of nematodes for 24 h, the WT strain could produce approximately 430 traps/cm^2^, twice as many as produced by the Δ*AfLaeA* strains (Fig. 5A). The traps of the WT strain became saturated after 24 h, whereas Δ*AfLaeA* strains required 36 h (Fig. 5A). Similarly, Δ*AfLaeA* strains required 96 h to capture all nematodes, whereas the WT strain required only 36 h (Fig. 5B). The WT strain produced two types of traps (regular and irregular) upon induction by nematodes (Fig. 5D), and both types of traps could catch and kill nematodes (Fig. 5E). Interestingly, the loss of the *AfLaeA* gene resulted in the appearance of more irregular (type II) traps, up to 34%, whereas about 1% of this type of trap was found in the WT strain (Fig. 5F). In addition, electron-dense bodies (EDs) in the trap cells of the WT and Δ*AfLaeA* strains were observed using TEM, and the results showed that fewer EDs were observed in the Δ*AfLaeA* strains than in the WT (Fig. 5G). Extracellular protease activity is also an indicator of fungal pathogenicity (38). Deletion of *AfLaeA* gene resulted in a significant decrease in extracellular protease activity, whereas overexpression of the *AfLaeA* gene resulted in enhanced activity (Fig. 5H). The WT strain showed approximately 3-fold-higher extracellular protease activity than the Δ*AfLaeA* strain, whereas the *OEAfLaeA* strains showed 1.5-fold-higher activity than the WT strain (Fig. 5H).

### Prediction and analysis of interactional proteins of AfLaeA.

**FIG 6:**
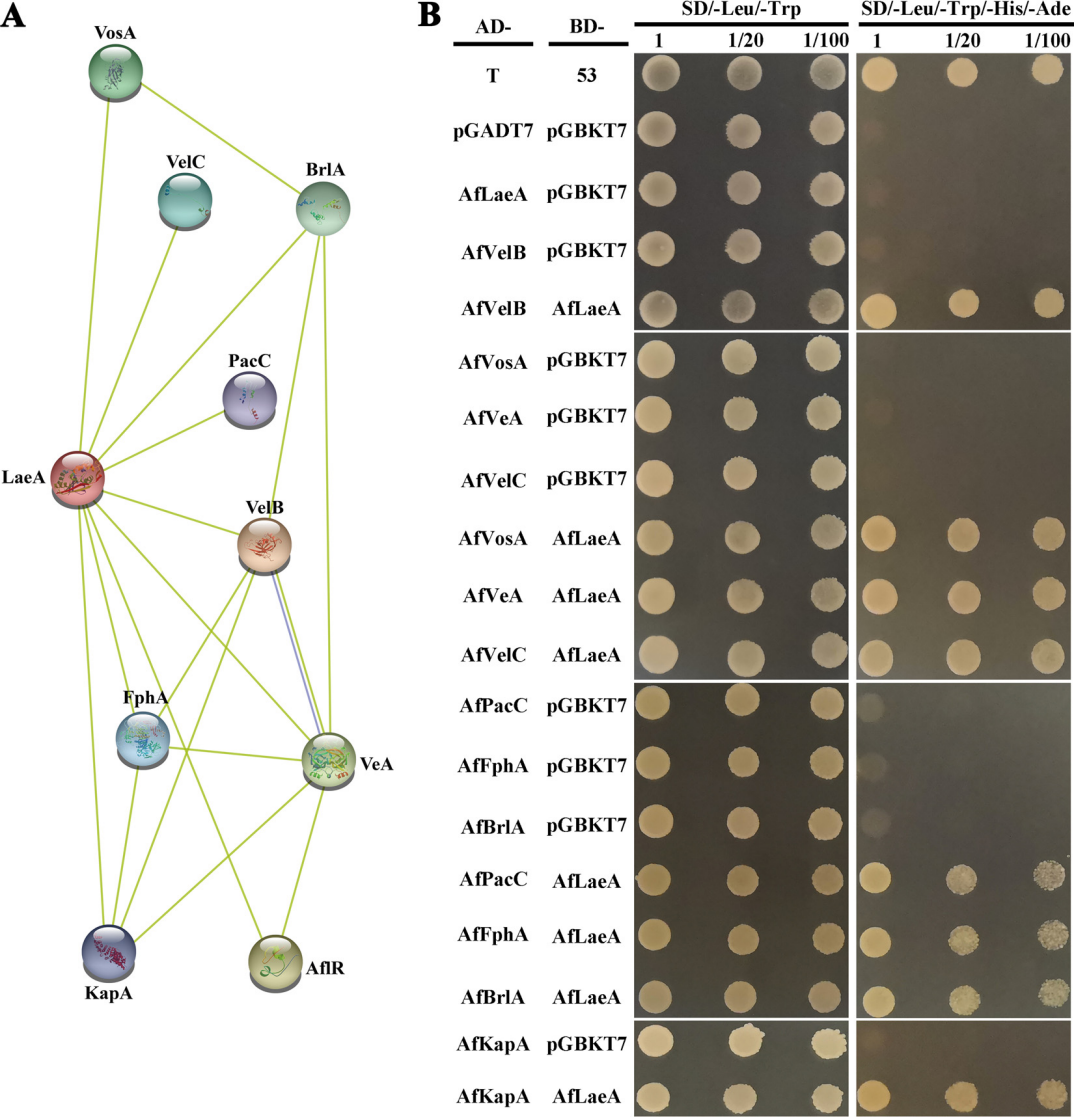
Analysis of the interacting protein with AfLaeA in the yeast system. (A) Prediction of LaeA-interacting networks using STRING software (http://string-db.org/). (B) Y2H analysis of the interactions between AfLaeA and four velvet family proteins (AfVosA, AfVeA, AfVelB, and AfVelC), AfPacC, AfFphA, AfBrlA, and AfKapA.

LaeA, as a methyltransferase, must interact with other proteins to form complexes, thereby regulating biological processes such as light response, secondary metabolism, growth, and development (1, 2, 11). In this study, we used AnLaeA as the query sequence and STRING online software to predict the interaction protein of LaeA. The proteins that interacted with AnLaeA included four velvet family proteins (VosA, VeA, VelB, and VelC), pH response transcription factor PacC, phytochrome FphA, C2H2 type master regulator BrlA, importin subunit alpha KapA, and sterigmatocystin biosynthesis regulatory protein AflR (Fig. 6A). With the exception of AflR, the other eight interaction proteins in *A. flagrans* could be predicted (Fig. 6B). Subsequently, this prediction was verified using the yeast two-hybrid (Y2H) assay, which showed that AfLaeA interacted with four velvet family proteins (AfVosA, AfVeA, AfVelB, and AfVelC), AfPacC, AfFphA, AfBrlA, and AfKapA (Fig. 6B). This result provided a good foundation for the subsequent experiments.

### Transcriptomic profile analysis of the WT and *AfLaeA* strains.

**FIG 7:**
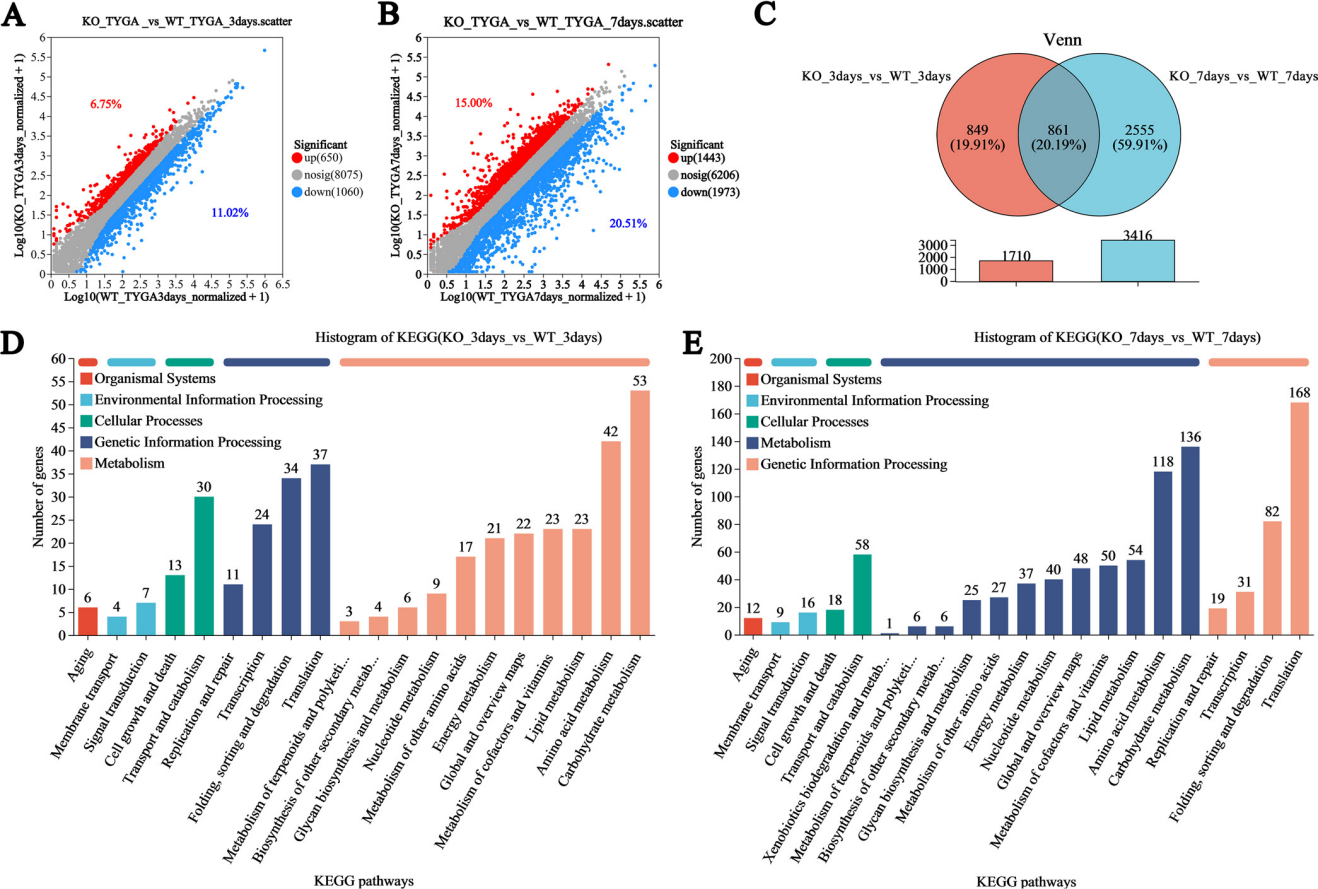
Transcriptomic insight into the regulatory role of AfLaeA (A and B) Differentially expressed genes (DEGs) on days 3 and 7, respectively. (C) Venn analysis of the DEGs at two time points in WT and Δ*AfLaeA* strains. (D and E) KEGG enrichment analysis of DEGs in WT and Δ*AfLaeA* strains.

To further investigate the regulatory mechanisms of AfLaeA in *A. flagrans*, the transcriptomic profiles of the WT and Δ*AfLaeA* strains were compared using transcriptome sequencing (RNA-seq). The results showed that the number of clean reads that could be located on the genome in each sample was more than 95% relative to that of the genome (Table S5). Principal-component analysis (PCA) showed that the WT and Δ*AfLaeA* strains were located in different quadrants at various time points, and the three duplicate samples had high similarity (Fig. S7). Δ*AfLaeA* strains were found to have 1,710 and 3,416 differentially expressed genes (DEGs) at 3 and 7 days, respectively (Fig. 7A and B). The number of downregulated and upregulated DEGs accounted for 11.20% and 6.75% and 20.51% and 15.00% at 3 days and 7 days, respectively (Fig. 7A and B). In addition, 849 and 2,555 DEGs were specifically expressed at 3 and 7 days, respectively (Fig. 7C).

On the third day, GO enrichment analysis showed that the GO terms most enriched by upregulated DEGs in the Δ*AfLaeA* strain were associated with the preribosome, nucleolus, and rRNA metabolic process, among others, whereas the downregulated DEGs were associated with the integral component of the membrane, the extracellular region, and the anchored component of the membrane, among others (Fig. S8A and B). On day 7, the upregulated GO terms included mitochondrial transport, tRNA aminoacylation for protein translation, and translational elongation, among others (Fig. S8C). Additionally, the downregulated GO terms included carbohydrate metabolic process, hydrolase activity, and DNA-binding transcription factor activity, among others (Fig. S8D). KEGG analysis showed that more DEGs were present in KEGG pathways on day 7, but the KEGG pathway types enriched for upregulated and downregulated genes, including lipid metabolism, energy metabolism, carbohydrate metabolism, aging, glycan biosynthesis and metabolism, cell growth and death, global and overview maps, signal transduction, folding, metabolism of terpenoids and polyketides, biosynthesis of other secondary metabolites, and nucleotide metabolism, among others, were the same on days 3 and 7 (Fig. 7D and E).

**FIG 8:**
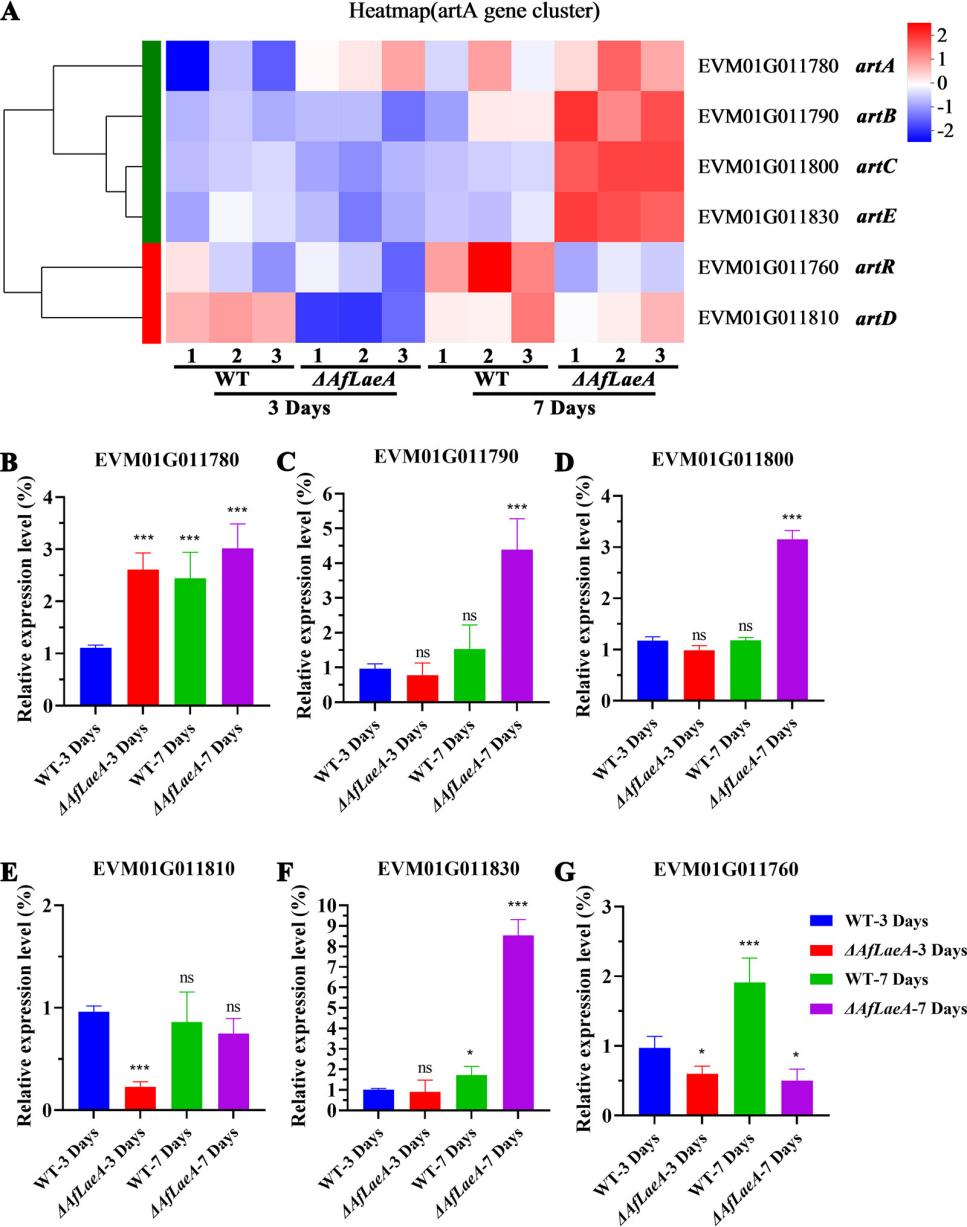
AfLaeA affects the expression of the *artA* gene cluster. (A) Transcriptome analysis of the expression level of the *artA* gene cluster at days 3 and 7. (B to G) The expression level of the *artA* gene cluster was analyzed by qPCR (ns, *P *> 0.05; *, *P *< 0.05; **, *P *< 0.01; ***, *P *< 0.001).

On the basis of the GO and KEGG analyses, we analyzed the *artA* gene cluster, which plays a key role in *A. flagrans* and *A. oligospora* (Fig. S9). The 6-methylsalicylic acid (6-MSA) produced by the *artA* gene cluster can fatally attract nematodes and regulate the production of traps in time and space (39). In this study, we found that the expression level of most genes in the *artA* gene cluster was low on day 3, but their expression level increased on day 7 (Fig. 8). The *artA* and *artD* genes had opposite expression patterns on day 3, but the patterns tended to converge by day 7 in the WT and Δ*AfLaeA* strains (Fig. 8A). Importantly, the expression level of the *artA* gene cluster in the Δ*AfLaeA* strain was found to be significantly higher than that of the WT strain, except for the *artR* gene (Fig. 8). Furthermore, the same results were obtained by quantitative real-time PCR (qPCR), as shown in Fig. 8B to G.

**FIG 9:**
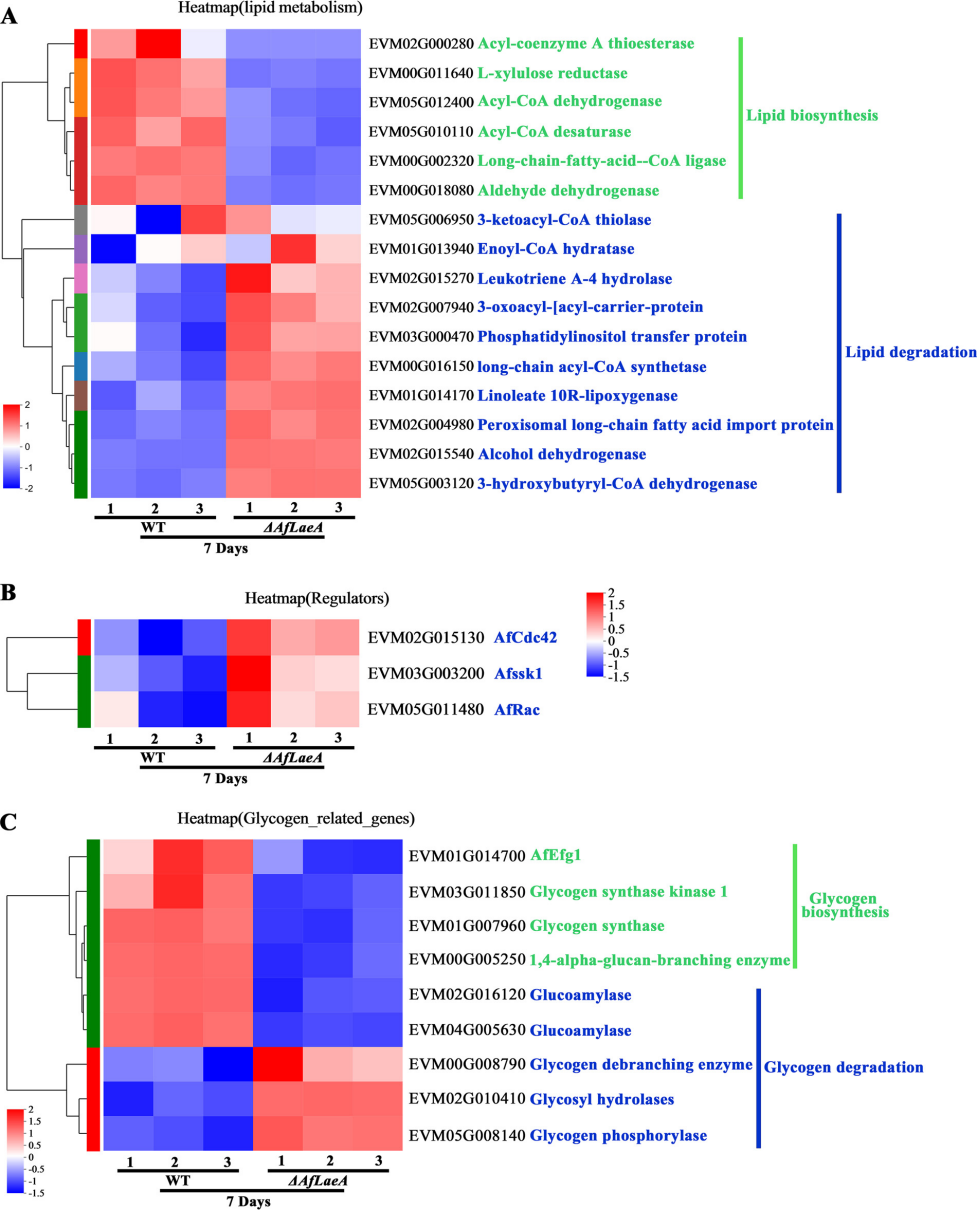
AfLaeA affects the expression of LDs and glycogen metabolism. (A) Transcriptome analysis of the expression level of LD metabolism genes at days 3 and 7. (B) Transcriptome analysis of the expression level of *AfCdc42*, *AfSSK1*, and *AfRac* at days 3 and 7. (C) Transcriptome analysis of the expression level of glycogen metabolism genes at days 3 and 7.

We analyzed several genes that were enriched in relation to lipid and glycogen metabolism. The DEGs involved in lipid synthesis and degradation showed different expression patterns in the WT and Δ*AfLaeA* strains (Fig. 9). Among the DEGs related to lipid metabolism in the Δ*AfLaeA* strains, six genes were involved in lipid biosynthesis (genes coding for acyl coenzyme A [acyl-CoA] thioesterase, acyl-CoA dehydrogenase, and acyl-CoA desaturase, among others), and 10 genes were involved in lipid degradation (genes coding for enoyl-CoA hydratase, long-chain acyl-CoA synthetase, and peroxisomal long-chain fatty acid import protein, among others) (Fig. 9A). In addition, the expression patterns of some regulatory genes (*AfCdc42*, *AfSSK1*, and *AfRac*) were also analyzed in the WT and Δ*AfLaeA* strains (Fig. 9B). The deletion of *AoCdc42*, *AoSSK1*, and *AoRac* genes led to accumulation of LDs in *A. oligospora* (40, 41). In this study, the deletion of *AfLaeA* led to upregulation of the expression levels of the *AfCdc42*, *AfSsk1*, and *AfRac* genes (Fig. 9B). Similarly, the effect of AfLaeA on glycogen production showed the same patterns, including glycogen biosynthesis and degradation (Fig. 9C). Glycogenin, glycogen synthase, and 1,4-α-glucan-branching enzymes are required for glycogen synthesis, while glycogen phosphorylase, glycogen debranching enzyme, glucoamylase, and glycosyl hydrolase are required for degradation. The glycogen biosynthesis-related genes were all less strongly expressed in Δ*AfLaeA* strains, but degradation-related genes were all more strongly expressed (Fig. 9C). In addition, *AfEfg1*, as a transcription factor that regulates the expression of glycogen synthase (42), also showed a lower expression level in Δ*AfLaeA* strains (Fig. 9C). Interestingly, two glycogen-degrading enzymes (glucoamylase) have high expression levels in the WT strain (Fig. 9C).

### Secondary metabolism insight into the regulatory role of AfLaeA in *A. flagrans*.

**FIG 10:**
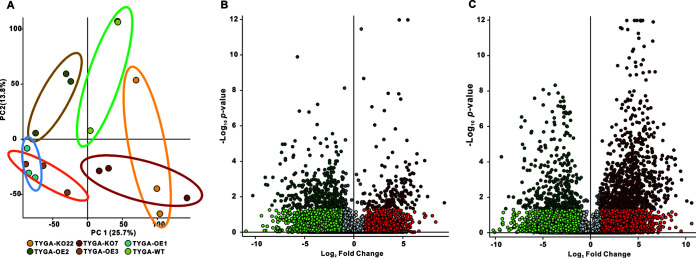
Comparison of metabolic profiles of fermentation broth from the WT, Δ*AfLaeA*, and OE strains. (A) PCA of the secondary metabolites produced by WT, Δ*AfLaeA*, and OE strains cultured in TYGA medium for 14 days. TYGA-WT represents the WT strain. TYGA-KO22 and TYGA-KO7 represent the Δ*AfLaeA* strains. TYGA-OE1, TYGA-OE2, and TYGA-OE3 represent the OE strains. (B) Volcano plot of differential metabolites in Δ*AfLaeA* strains compared with the WT strain. The 340 compounds on the left half of the plot were present at significantly higher levels in the WT sample. (C) Volcano plot of differential metabolites in *OEAfLaeA* strains compared with the WT strain. The 860 compounds on the right half of the plot are present at significantly higher levels in the OE-3 sample.

Regulation of secondary metabolism is one of the main functions of LaeA in fungi (6). In this study, to investigate the effect of LaeA on secondary metabolism, the WT, Δ*AfLaeA*, and OE strains were cultured in TYGA medium for 14 days. Subsequently, we used liquid chromatography-mass spectrometry (LC-MS) and untargeted metabolomics to evaluate the function of AfLaeA involved in secondary metabolite regulation in *A. flagrans*. PCA showed that the secondary metabolites produced by the WT, Δ*AfLaeA*, and OE strains were independently distributed in the quadrants (Fig. 10A). Under the screening conditions of a *P* value of <0.05 and a fold change difference of >2, we observed 171 upregulated and 340 downregulated metabolites in Δ*AfLaeA* strains (Fig. 10B), while 860 upregulated and 353 downregulated metabolites were found in the OE-3 strain (Fig. 10C). Among these differential metabolites, the most downregulated (564.56-fold) compound was found at an *m/z* of 331.2378 with a retention time of 17.007 min in Δ*AfLaeA* strains, while the most upregulated (1,204.756-fold) compound in the OE-3 strain was found at an *m/z* of 445.2958 with a retention time of 17.186 min (Table S6). The top 20 downregulated and unregulated metabolites of the Δ*AfLaeA* and OE strains are listed in Table S6. According to the chromatogram, deletion of *AfLaeA* resulted in the disappearance of several metabolites, but several new compounds appeared (Fig. S10). The major missing secondary metabolites in Δ*AfLaeA* strains reappeared, and several new secondary metabolites were produced by overexpression of *AfLaeA* (Fig. S10).

## DISCUSSION

In fungi, several global regulators of secondary metabolism, including mitochondrial complex I, SaraC, and LaeA, have been reported (43, 44). To date, LaeA has been identified mainly in multiple ascomycetes but recently also in basidiomycetes (see Table S7 in the supplemental material). Consistently, LaeA has crucial functions in secondary metabolism and fungal development in most ascomycetes (6, 45) in addition to involvement in the pathogenicity of multiple pathogenic fungi (Table S7). As for the pathogenic fungi of animal/plant-parasitic nematodes, several genes, such as the *SofT* gene (32), the *artA* gene cluster (39), and genes coding for small secreted cysteine-rich protein CyrA (37) and STRIPAK (striatin-interacting phosphatase and kinase) component SipC (46), are involved in fungal development and virulence toward nematodes in *A. flagrans*. However, the function of LaeA remains poorly understood in NT fungi.

### LaeA is required for mycelial growth of *A. flagrans*.

Previous studies have shown that deletion of LaeA gene leads to slow growth of *Trichoderma* spp. (8), *C. heterostrophus* (17), Pestalotiopsis microspora (47), and Coprinopsis cinerea (48). In the present study, the colony growth of Δ*AfLaeA* strains was remarkably smaller than that of the WT strain (Fig. 3). In contrast, this phenotypic change in Δ*AfLaeA* strains did not coincide with those reported in *M. ruber*, whose *MrLaeA* mutants formed abnormal colonies that had abundant aerial hyphae with a higher growth rate than that of the WT (21). Interestingly, loss of *AnVelB* led to a lower growth rate than that of the WT in A. nidulans (49). In addition, the Δ*AoVelB* mutants showed significant growth defects on PDA, TYGA, and TG media in terms of *A. oligospora* (50). Importantly, the different combinations of LaeA and velvet family proteins have different biological functions, such as tight regulation of the morphogenesis of filamentous fungi (1, 19, 20), and these interactions were also confirmed in *A. flagrans* using Y2H assays (Fig. 6). This finding indicates that LaeA and VelB are required for mycelial growth with the same function and that hyphal growth may be regulated by LaeA and VelB complexes.

### LaeA is a key regulator of chlamydospore production in *A. flagrans*.

A chlamydospore is a thick-walled hypopus produced by fungal hyphae under adverse environmental conditions (low temperature, unfavorable pH, and nutrient deficiency, among others), which can help fungi adapt to harsh environments (51). It has been reported that many fungi, such as the unicellular eukaryotes Candida albicans, Candida dubliniensis, and Cryptococcus laurentii (52–54), filamentous fungi F. oxysporum, Trichoderma harzianum, and Metarhizium anisopliae (55, 56), and the macrofungus Coprinus cinereus (57) can produce chlamydospores during the morphological differentiation stage. In the unicellular eukaryote C. albicans, *Hog1*, *cek1*, *cla4*, *cst20*, *hst7*, *cph1*, *cpp1*, *Gcn4*, *Gln3*, *NCR*, *Gat1*, *Nrg1*, *Efg1*, *RME1*, *Dpm1*, and *Dpm3* genes are involved in the formation of chlamydospores (58, 59). In addition, the chlamydospores of F. oxysporum and *T. reesei* are positively regulated by calcineurin gene *CNA1* and chitin synthase genes *Chs1_7926* and *Chs1_8917*, respectively (60, 61). Notably, deletion of the methyltransferase *phcR* gene in Ralstonia solanacearum leads to an increase in chlamydospores (62). In this study, deletion of *AfLaeA* gene abolished the capability of *A. flagrans* to produce chlamydospores (Fig. 4).

A lack of the STRIPAK component SipC results in a reduced number of conidia and chlamydospores, and the morphology of 71.3% chlamydospores in mutants is also greatly expanded (46). SipC, as a highly conservative eukaryotic signal center, is necessary for the asexual development of *A. flagrans* and plays an important role in the determination of cell fate (46). Interestingly, the STRIPAK complex is required for the regulation of fungal development and the correct expression of the secondary metabolic heterotrimeric VeA-VelB-LaeA complex in A. nidulans (63). In Ustilaginoidea virens, chlamydospore formation is regulated by *UvAtg7*, *UvHOX2*, and *UvVEA*. Loss of *UvAtg7* and *UvHOX2* genes was found to lead to defects in chlamydospore production (64, 65), and loss of the *UvVEA* gene led to a decrease in the number of chlamydospores (66). A STRING website prediction and Y2H analysis showed that AfLaeA can interact with AfVeA (Fig. 6), but the biological function of AfVeA in *A. flagrans* is still unknown. The interaction between LaeA and other proteins can strictly regulate the fungal development of Aspergillus spp. and many other fungi (Table S7). These evidences provide the basis for further understanding of the formation mechanism of chlamydospores.

### LaeA affects the energy metabolism in *A. flagrans*.

Apart from the abolished capacity of chlamydospore production, LaeA affects the energy metabolism of *A. flagrans*, including glycogen and lipid metabolic processes (Fig. 4E). Lipids and glycogen are the main energy storage material of conidia and chlamydospores (42). These energy substances can be degraded during spore germination to provide the energy needed for fungal growth and initial materials for synthesizing other cellular components (67). In this study, deletion of *AfLaeA* resulted in a reduction in glycogen and lipid accumulation in mycelium (Fig. 4E). In addition, our transcriptomic analysis demonstrated that 21 and 37 DEGs were annotated to the energy metabolism pathway, whereas 23 and 54 DEGs were annotated to the lipid metabolism pathway, at days 3 and 7, respectively (Fig. 7D and E). The expression levels of genes related to lipid and glycogen synthesis and degradation showed opposite trends in the WT and Δ*AfLaeA* strains (Fig. 9). In terms of lipid metabolism, several genes involved in lipid degradation were upregulated in Δ*AfLaeA* strains (Fig. 9), and two genes (those encoding enoyl-CoA hydratase and long-chain acyl-CoA synthetase) are involved in the β-oxidation cycle (Fig. 9A), which constitutes the major pathway of fatty acid degradation in most fungi (37). This result indicates that the degradation of fatty acids is enhanced in Δ*AfLaeA* strains. In addition, two Rho GTPase genes (*Rac* and *Cdc42*) and *Ssk1* play an important role in lipid accumulation in *A. oligospora* (40, 41). In this study, regulatory genes (*AfCdc42*, *AfSsk1*, and *AfRac*) had higher expression levels in Δ*AfLaeA* strains (Fig. 9B).

In terms of glycogen metabolism, glycogen synthesis is controlled by protein kinase A signaling via the transcription factor *CaEfg1* in C. albicans. Loss of *CaEfg1* results in a decrease in intracellular glycogen content (42). In this study, loss of the *AfLaeA* gene resulted in a reduction in glycogen accumulation (Fig. 4E) and a reduction in expression levels of *AfEfg1* and glycogen synthase (Fig. 9C). In contrast, loss of glycogen synthase *YlGSY1* was found to lead to a decrease in glycogen but an increase in lipid content in oleaginous Yarrowia lipolytica. Glycogen synthesis is a competing pathway for lipid accumulation in oleaginous yeasts (68). In *A. flagrans*, glycogen accumulation and lipid accumulation occur concurrently (Fig. 4), which also illustrates the similarities and differences of energy metabolism in different species.

### AfLaeA strongly regulates the secondary metabolism of *A. flagrans*.

LaeA regulates the expression of multiple secondary metabolic gene clusters and affects the formation of secondary metabolites. Loss of the LaeA gene hindered the expression of multiple secondary metabolic gene clusters, including kojic acid, cyclopiazonic acid, citrinin, citric acid, and the lovastatin gene cluster (2, 30, 69–71). In *Valsa mali*, about half (31/60) of the secondary metabolism gene clusters are regulated by *VmLaeA* (15). Similarly, most PKS and NRPS genes were differentially regulated by *Lae1* in *T. reesei* (72). In this study, our results showed that deletion of *AfLaeA* gene resulted in upregulation of most of the genes (*artA*, *artB*, *artC*, and *artE*) in the *artA* cluster (Fig. 8). It was previously reported that arthrosporols produced by the PKS gene cluster (*artA*) can inhibit trap production in *A. flagrans* (39). Interestingly, under induction by nematodes, trap production was much slower in Δ*AfLaeA* strains, and the number of traps was reduced approximately 50% compared with that in the WT (Fig. 5A). This result may be due to the upregulation of some *artA* cluster genes.

In addition, it has been commonly observed that the overexpression of LaeA has been successfully used to activate some gene clusters, enhance production of several secondary metabolites, and produce new compounds in fungi (45). Overexpression of LaeA increases the transcription and product formation of genes involved in the kojic acid antitumor compounds terrequinone A, cyclopinionic acid, and tertrimilone (2, 30, 69–72). Moreover, overexpression of LaeA results in the production of the novel compound in Aspergillus versicolor 0312 (73), Chaetomium globosum (29), and Penicillium dipodomyis YJ-11 (74), respectively. In *A. flagrans*, both deletion and overexpression of *AfLaeA* gene led to significant changes in secondary metabolites. Deletion of *AfLaeA* gene led to downregulation of 340 metabolites, while overexpression of *AfLaeA* led to upregulation of 860 metabolites (Fig. 10). Furthermore, deletion or overexpression of *AfLaeA* gene resulted in the loss of multiple secondary metabolites in addition to the production of multiple new compounds (Fig. S10).

### AfLaeA is associated with the pathogenicity of *A. flagrans*.

LaeA has been shown to positively regulate pathogenicity toward the host in several pathogenic fungi (14–17). The pathogenicity of these pathogenic fungi was quantified based on the degree of colonization in the host and virulence factors such as mycotoxins and serine proteases (50). However, the pathogenicity of NT fungi is demonstrated by the number of traps, the capability to capture nematodes, and the activity of virulence factors, such as small secreted proteins and serine proteases (37, 50). In the present study, deletion of *AfLaeA* gene resulted in a delay in trap generation, an increased proportion of irregular traps, and a reduction in the number of traps (Fig. 5). The reason for this phenomenon is estimated to be related to the regulation of fungal growth and the *artA* cluster by AfLaeA. Additionally, compared with that of the WT strain, the proteolytic activity of Δ*AfLaeA* strains was weakened but that of *OEAfLaeA* strains was enhanced (Fig. 5H). It was previously reported that the loss of LaeA resulted in strong underexpression of infection-related proteins in Botrytis cinerea (75), reduction of extracellular proteins in Beauveria bassiana (76), and decreases of extracellular hydrolases in A. flavus (77). After the potential interacting protein VelB of LaeA was disrupted, the extracellular proteolytic activities were reduced, and the transcriptional levels of five serine protease genes were downregulated in *A. oligospora* (50).

### The function of LaeA is epigenetic regulation of downstream genes.

Epigenetic mechanisms control the expression of genes in chromosomal regions by regulating their state. When condensed and tightly packed heterochromatin is formed, genes are silenced, whereas when relaxed euchromatin is formed, genes can be transcribed for function (78). LaeA contains a SAM binding domain and appears to methylate histone proteins differentially to alter the chromatin structure for promoting gene expression (1). In a previous report, the SAM domain of BcLAE1 was shown to be involved in abscisic acid biosynthesis in *B. cinerea* TB-31 (45). However, in *A. flagrans*, the key active domain of AfLaeA should not be the SAM domain because deletion of the two methyltransferase genes with the same SAM domain has no effect on mycelial growth or chlamydospore production (Fig. S4 and S6). In Aspergillus spp., the expression of LaeA was negatively regulated by the Ras transduction pathway and cyclic AMP/protein kinase A (cAMP-PKA signaling pathway), which can respond to environmental stimuli (2). In terms of growth in such a scenario, an LaeA-mediated process would trigger the removal of heterochromatic marks, thus allowing the transcription of the downstream genes (79). Similarly, when chlamydospores are produced under unfavorable environmental conditions, the production mechanism of chlamydospores may be regulated via a similar signaling pathway involving LaeA.

## MATERIALS AND METHODS

### Fungal strains and culture conditions.

*A. flagrans* CBS 565.50 was stored in the Microbial Library of the Germplasm Bank of Wild Species from Southwest China. *A. flagrans* was cultivated on PDA (Sigma) medium at 28°C. The mutants (Δ*AfLaeA*, Δ*DFL_000451*, Δ*DFL_006623*, and Δ*DFL_007594*) were cultivated on PDA medium containing 100 μg/mL hygromycin B. The overexpression strains (*OEAfLaeA*) were cultivated on PDA medium containing 100 μg/mL hygromycin B and 50 μg/mL nourseothricin.

### DNA and RNA isolation and gene cloning.

The total genomic DNA was extracted from 4-day-old cultured fungal hyphae using a HiPure fungal DNA kit II (Magen). Extraction of total RNA and first-strand cDNA synthesis were performed using RNA-easy isolation reagent (Vazyme) and a HiScript III first-strand cDNA synthesis kit (+gDNA wiper) (Vazyme), respectively. Genomic DNA and cDNA were used for PCR amplification of the genes or DNA fragments using specific primers (see Table S8 in the supplemental material). Finally, the PCR products were used for agarose gel electrophoresis and sequencing analysis.

### Chromosome-level genome assembly and annotation.

Fifteen micrograms of DNA was used to construct a PacBio Sequel reads library by using a SMRTbell express template prep kit 2.0 (PacBio), and PacBio Sequel II technology was used to sequence the whole genome circular consensus sequencing (CCS). The sequencing runs and assembly of the libraries were performed by Biomarker Technologies (China), and the genome analysis was performed using BMKCloud (https://www.biocloud.net).

### Sequence and phylogenetic analysis.

The DNA sequences of *AfLaeA* and the methyltransferase gene were extracted from the genome data of *A. flagrans* CBS 565.50. The gene structure of *AfLaeA* was analyzed using the online software Gene Structure Display Server (http://gsds.cbi.pku.edu.cn/). MEME (Multiple Expectation Maximization for Motif Elicitation; http://meme-suite.org/), SMART (Simple Modular Architecture Research Tool; http://smart.embl.de), and NCBI online tool Conserved Domain Search Service (https://www.ncbi.nlm.nih.gov/Structure/cdd/wrpsb.cgi) were used to identify and annotate the conserved SAM binding site (2). BioEdit software was used for sequence alignment, and the software package MEGA X (http://www.megasoftware.net/) was used for phylogenetic analysis. NCBI’s Basic Local Alignment Search Tool (https://blast.ncbi.nlm.nih.gov/Blast.cgi) was used to analyze the similarity of proteins. In addition, the subcellular localization of proteins was predicted by Euk-mPLoc 2.0 software (http://www.csbio.sjtu.edu.cn/bioinf/euk-multi-2/).

### Plasmid construction.

The *AfLaeA* gene was knocked out by the homologous recombination method. In brief, two fragments of 1,036 bp and 960 bp corresponding to the 5′ and 3′ regions of the *AfLaeA* gene, respectively, were amplified using PCR with primer sets ko5107-5-for/rev and ko5107-3-for/rev (Table S8), respectively, from *A. flagrans* genomic DNA. The hygromycin B resistance cassette (*hph*) was amplified using PCR with primers (Table S8) from the pCSN44 vector. All fragments were then assembled in the pUC19 vector (digested with EcoRI and NdeI) by using the ClonExpress Ultra one-step cloning kit (Vazyme). Subsequently, the gene knockout fragment of the *AfLaeA* gene was amplified by PCR with primers ko5107-5-for and ko5107-3-rev and recovered to a concentration of 5 to 10 μg/μL. The gene knockout plasmid of three methyltransferase genes (*DFL_000451*, *DFL_006623*, and *DFL_007594*) were assembled by the same method (Fig. S2).

For overexpression, 2,000- and 600-bp PCR products containing the promoter and downstream regions of the glyceraldehyde-3-phosphate dehydrogenase (*AfGpd*) gene, respectively, were amplified using the specific primers (Table S8). The 720-bp enhanced green fluorescent protein (EGFP) gene was amplified from the pCT74 vector via PCR. In addition, a 1,086-bp PCR product containing the full-length *AfLaeA* coding region was amplified from *A. flagrans* cDNA, and a nourseothricin resistance cassette (*Nrs^r^*) was amplified from the pCfB3052 vector. All fragments were sequentially assembled into the pUC19 vector (digested with EcoRI and NdeI) by using the ClonExpress Ultra one-step cloning kit (Vazyme).

For complementation, the experiment was completed using the native promoter of the *AfLaeA* gene to complement the *AfLaeA* gene in the mutant strain. In brief, a 1,086-bp PCR product containing the *AfLaeA* coding region and 2.0-kb upstream and 1.0-kb downstream regions was amplified using the specific primers (Table S8). Similarly, the nourseothricin resistance cassette (*Nrs^r^*) was amplified from the pCfB3052 vector, and all fragments were sequentially assembled into the pUC19 vector by the same method as used for overexpression.

### Protoplast transformation of *A. flagrans*.

The transformation system was adapted as previously described (38). In brief, 1 × 10^8^ chlamydospores were inoculated into 100 mL of potato dextrose broth (PDB) and incubated at 28°C and 180 rpm for 24 h. Subsequently, the mycelium was harvested and washed with STC buffer (1 M sorbitol, 50 mM CaCl_2_, 10 mM Tris-HCl), and approximately 1 g of mycelium (wet weight) was collected and suspended in 2 mL MN buffer (0.3 M MgSO_4_, 0.3 M CaCl_2_) containing 1 mg/mL lysing enzyme (Sigma), 2 mg/mL Snailase (Solarbio), and 2 mg/mL cellulase (Solarbio), followed by incubation at 28°C and 90 rpm for 4 h. Subsequently, the protoplasts were filtered using six layers of lens wiping paper and precipitated at 3,000 × *g* for 10 min. The protoplasts were washed with 10 mL of sorbitol-Tris-calcium chloride (STC) buffer twice, and 200 μL of protoplasts (about 2 × 10^8^) was left at the bottom of the tube, after which 6 to 10 μg of DNA fragment was mixed into 100 μL of protoplasts and incubated on ice for 30 min. One milliliter of PTC (10 mM Tris-HCl [pH 7.5], 50 mM CaCl_2_, 50% [wt/vol] polyethylene glycol [PEG] 3350) was added and incubated at 28°C for 40 min. Finally, PDSSA medium (24 g/L potato dextrose broth, 0.6 M sucrose, 0.3 g/L peptone, 0.3 g/L tryptone, 0.3 g/L yeast extract, 8 g/L agar) was added to the transformation samples. After the petri dishes were incubated at 28°C for 2 days, the PDA medium containing 100 μg/mL hygromycin B or 50 μg/mL nourseothricin was added to the petri dishes to screen for the positive strains.

### Comparison of mycelial growth and analysis of stress tolerance.

WT and Δ*AfLaeA* strains (6 mm in diameter) were cultivated on PDA, TG (1% tryptone, 1% glucose, 1.5% agar), and TYGA (1% tryptone, 0.5% yeast extract, 1% glucose, 0.5% molasses, 1.5% agar) media for 5 days at 28°C, and the colony diameters were measured daily. For stress tolerance analysis, WT and Δ*AfLaeA* strains (6 mm in diameter) were cultivated on PDA medium containing chemical stress reagents at different concentration for 5 days at 28°C. In brief, osmotic pressure stress reagent NaCl (0.1, 0.2 and 0.3 M) and cell wall stress reagent SDS (0.01% to 0.03%) were added to the PDA medium. All the experiments were performed at least three times, and the relative growth inhibition (RGI) was calculated using the diameter of each colony.

### Trap formation and pathogenicity assays.

For traps, after WT and Δ*AfLaeA* strains (6 mm in diameter) were cultivated on water agar (WA) medium (2% agar–water) for 3 days at 28°C, approximately 400 to 600 second larval stage (L2) of C. elegans was added to the petri dishes (6 cm in diameter) to induce trap formation, and after the addition of nematodes for 3 h, the trap formation and number of captured nematodes were observed until 96 h. The shape and number of traps were recorded using a microscope and SEM. For extracellular protease activity, the WT, Δ*AfLaeA*, and *OEAfLaeA* strains (6 mm in diameter) were inoculated into 100 mL of LMZ medium [gelatin, 20 g/L; peptone, 8 g/L; yeast extract, 1 g/L; (NH_4_)_2_SO_4_, 0.5g/L; MgSO_4_, 0.5g/L; FeSO_4_, 0.01 g/L] for 7 days at 180 rpm and 28°C. Ten percent skim milk was added to the WA medium to generate the skim milk dishes, and a puncher was used to prepare the 6-mm hole. Then, 200 μL of fermentation broth of the WT, Δ*AfLaeA*, and *OEAfLaeA* strains and TYGA liquid medium were added to the holes. Subsequently, the petri dishes were store at 37°C for 24 h, and the hydrolysis circles were observed under a microscope (Carl Zeiss, Germany).

### Cell wall, lipid, glycogen, and nucleic acid staining.

As previously described (32), calcofluor white (Sigma) (20 μg/mL) and 4′,6-diamidino-2-phenylindole (DAPI) (20 μg/mL) were used to stain the cell wall and nuclei, respectively. Glycogen of *A. flagrans* hyphae and chlamydospores was stained with Lugol’s iodine (Sigma) for 3 min. In addition, LDs were stained with 30 μL of 10 μg/mL BODIPY staining solution (Sigma) for 30 min. Subsequently, the samples were washed twice with phosphate-buffered saline (PBS). The pictures were observed under a fluorescence microscope (Carl Zeiss, Germany).

### Real-time fluorescent quantitative PCR assay.

Real-time fluorescent quantitative PCR was performed using AceQ qPCR SYBR green master mix (Vazyme) on a Roche LightCycler 480 system (Roche Applied Science) with the gene-specific primer pairs (Table S9). The amplification conditions were 94°C for 5 s and 60°C for 30 s for 40 cycles. The glyceraldehyde-3-phosphate dehydrogenase (*AfGpd*) gene was used as an internal control, and the 2^−ΔΔ^*^CT^* method was used to calculate the relative transcription level. All assays were repeated at least three times.

### Y2H assay.

In order to confirm the interaction between AfLaeA and the predicted proteins, the Y2H assay was used in this study. According to the manufacturer’s instructions (Clontech), the cDNA fragments of *AfLaeA* were inserted into the pGBKT7 plasmid to generate bait vectors (*AfLaeA*-BD), whereas the *AfVosA*, *AfVeA*, *AfVelB*, *AfVelC*, *AfPacC*, *AfFphA*, *AfBrlA*, and *AfKapA* cDNA fragments were inserted into the pGADT7 plasmid to generate the prey vectors *AfVosA*-AD, *AfVeA*-AD, *AfVelB*-AD, *AfVelC*-AD, *AfPacC*-AD, *AfFphA*-AD, *AfBrlA*-AD, and *AfKapA*-AD, respectively. These plasmids were transformed into AH109 yeast cells using a yeast transformation kit (Coolaber), plating on synthetic dropout medium lacking Trp and Leu (SD/–Trp/–Leu), and incubation for 3 days at 30°C. BD-53 and AD-T vector pairs were transformed into AH109 yeast cells to generate positive controls, whereas the empty BD and AD vectors were used as negative controls. The positive yeast cells were selected on SD/–Trp/–Leu medium and transferred to SD/–Trp/–Leu/–His/–Ade medium with serial dilutions (1, 1/20, and 1/100) to determine protein interactions between different pairs.

### LC-MS assays.

The secondary metabolites of WT and Δ*AfLaeA* strains were analyzed by an LC-MS assay in this study. WT and Δ*AfLaeA* strains were cultured using TYGA liquid medium for 7 days, respectively. The fermentation broths were collected by filtration using a vacuum filter pump. Then, the fermentation broths were extracted three times by mixing them with the same volume of ethyl acetate. The extracts were evaporated under vacuum and dissolved in chromatography-grade methanol (SK, Korea). Finally, the samples were filtered through a 0.22-μm filter and subjected to LC-MS (Thermo Scientific Ultimate 3000; Thermo Fisher Scientific, USA). The metabolic profiles of the WT and Δ*AfLaeA* strains were compared using Thermo Xcalibur software (Thermo Fisher Scientific). Untargeted metabolomics was performed using Compounds Discoverer 3.0 software (Thermo Fisher Scientific).

### SEM and TEM assays.

In this study, SEM was used to observe the differences in morphology, chlamydospores, and traps between WT and Δ*AfLaeA* strains, while TEM was used to observe the differences in the mycelial interior structure (LDs and EDs) at the hyphal stage, the chlamydospore-producing stage, and the chlamydospore, respectively. In order to analyze the differences in morphology, WT and Δ*AfLaeA* strains were cultivated on PDA medium with the dialysis membrane (9 cm in diameter) on the surface for 4 days at 28°C. To observe the capacity of chlamydospore production, WT and Δ*AfLaeA* strains were cultivated on WA medium (2% agar–water) for 8 days at 28°C. For the SEM assay, the samples were fixated with 4% glutaraldehyde for 20 min and dehydrated using an ethanol gradient (70%, 80%, 90%, and 100%). Then, isoamyl acetate (Sigma) was used to treat the sample for 10 min. After drying with liquid carbon dioxide, the samples were observed by SEM. For the TEM assay, the samples were fixed with 2.5% glutaraldehyde and stored at 4°C for at least 12 h. Afterward, the samples were observed under a TEM.

### Transcriptome sequencing and analysis.

To compare the effects of the *AfLaeA* gene on the expression of related genes in *A. flagrans*, WT and Δ*AfLaeA* strains were cultured using TYGA liquid medium at 28°C for 3 and 7 days, respectively. Then, the samples were collected and frozen in liquid nitrogen and stored at −80°C. Sequencing of mycelial samples was performed by Shanghai Majorbio Bio-pharm Technology Co., Ltd. (Shanghai, China), and the data were analyzed by the Majorbio Cloud Platform (https://www.majorbio.com). DESeq2 software was used to analyze the DEGs, and the analysis of the conditions of the DEGs included up/down differential multiples of >2 and an adjusted *P* value of <0.05.

### Statistical analyses.

In this study, all experiments were repeated at least three times, and statistical analyses were performed using GraphPad Prism v8.3. The data are expressed as mean values ± standard deviations. One-way analysis of variance, followed by Tukey’s test, was performed to determine significance.

### Data availability.

All data generated or analyzed during this study are included in the published paper and the associated supplemental files. The genes numbered “DFL_” and “EVM” correspond to the *Duddingtonia* (*Arthrobotrys*) *flagrans* genome of BioProject accession no. PRJNA494930 and PRJNA917252, respectively. The RNA-seq data presented here are associated with NCBI BioProject PRJNA970849 and BioSample SAMN34997886. The whole-genome shotgun project was deposited in GenBank under BioProject accession no. PRJNA917252 and BioSample accession no. SAMN32532839.
