# Supplementary material for: AfLaeA, a Global Regulator of Mycelial Growth, Chlamydospore Production, Pathogenicity, Secondary Metabolism, and Energy Metabolism in the Nematode-Trapping Fungus Arthrobotrys flagrans
Source: Microbiol Spectr. 2023 Jun 26;11(4):e00186-23. doi: 10.1128/spectrum.00186-23 (PMC10434191; doi:10.1128/spectrum.00186-23)
Supplement: Supplemental file 1 — Supplemental material. Download spectrum.00186-23-s0001.pdf, PDF file, 1.7 MB [file spectrum.00186-23-s0001.pdf]

Supplementary materials for

AfLaeA, a Global Regulator of Mycelial Growth, Chlamydospore Production, Pathogenicity, Secondary Metabolism, and Energy Metabolism in the Nematode-Trapping Fungus *Arthrobotrys flagrans*

Yu Zhang, Xin Wang, Yuan Ran, Ke-Qin Zhang, Guo-Hong Li*

State key Laboratory for Conservation and Utilization of Bio-Resources in Yunnan, School of Life Sciences, Yunnan University, Kunming, Yunnan 650091, China.

*Correspondence and requests for materials should be addressed to Guo-Hong Li (ligh@ynu.edu.cn).

Content

1. Figure S1-S10.

2. Table S1-S9

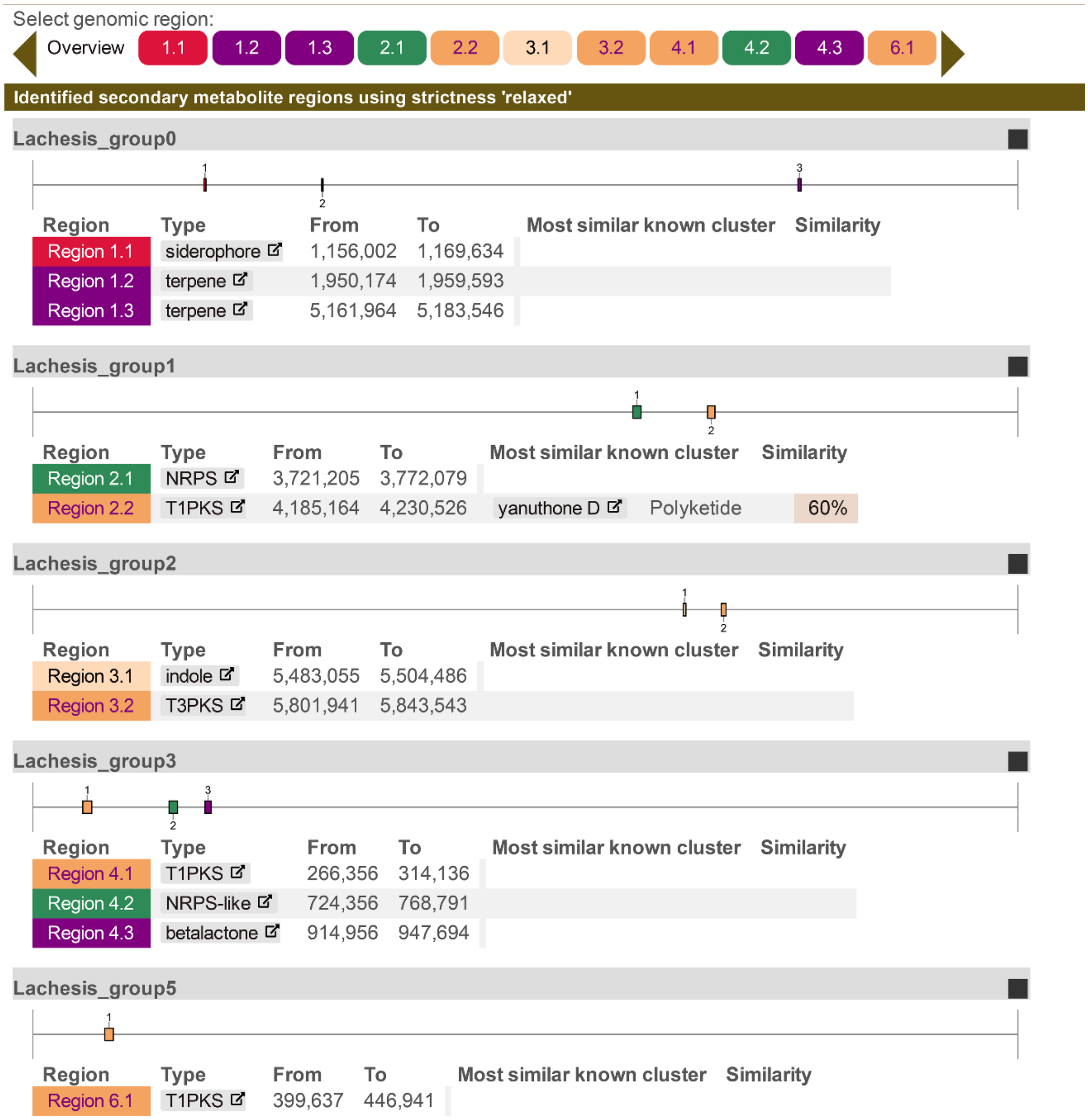

Lachesis_group0

Region	Type	From	To	Most similar known cluster		Similarity
Region 1.1	siderophore	1,156,002	1,169,634			
Region 1.2	terpene	1,950,174	1,959,593			
Region 1.3	terpene	5,161,964	5,183,546			

Lachesis_group1

Region	Type	From	To	Most similar known cluster		Similarity
Region 2.1	NRPS	3,721,205	3,772,079			
Region 2.2	T1PKS	4,185,164	4,230,526	yanuthone D	Polyketide	60%

Lachesis_group2

Region	Type	From	To	Most similar known cluster		Similarity
Region 3.1	indole	5,483,055	5,504,486			
Region 3.2	T3PKS	5,801,941	5,843,543			

Lachesis_group3

Region	Type	From	To	Most similar known cluster		Similarity
Region 4.1	T1PKS	266,356	314,136			
Region 4.2	NRPS-like	724,356	768,791			
Region 4.3	betalactone	914,956	947,694			

Lachesis_group5

Region	Type	From	To	Most similar known cluster		Similarity
Region 6.1	T1PKS	399,637	446,941			

Figure S1 Prediction of secondary metabolites gene clusters in *A. flagrans.*

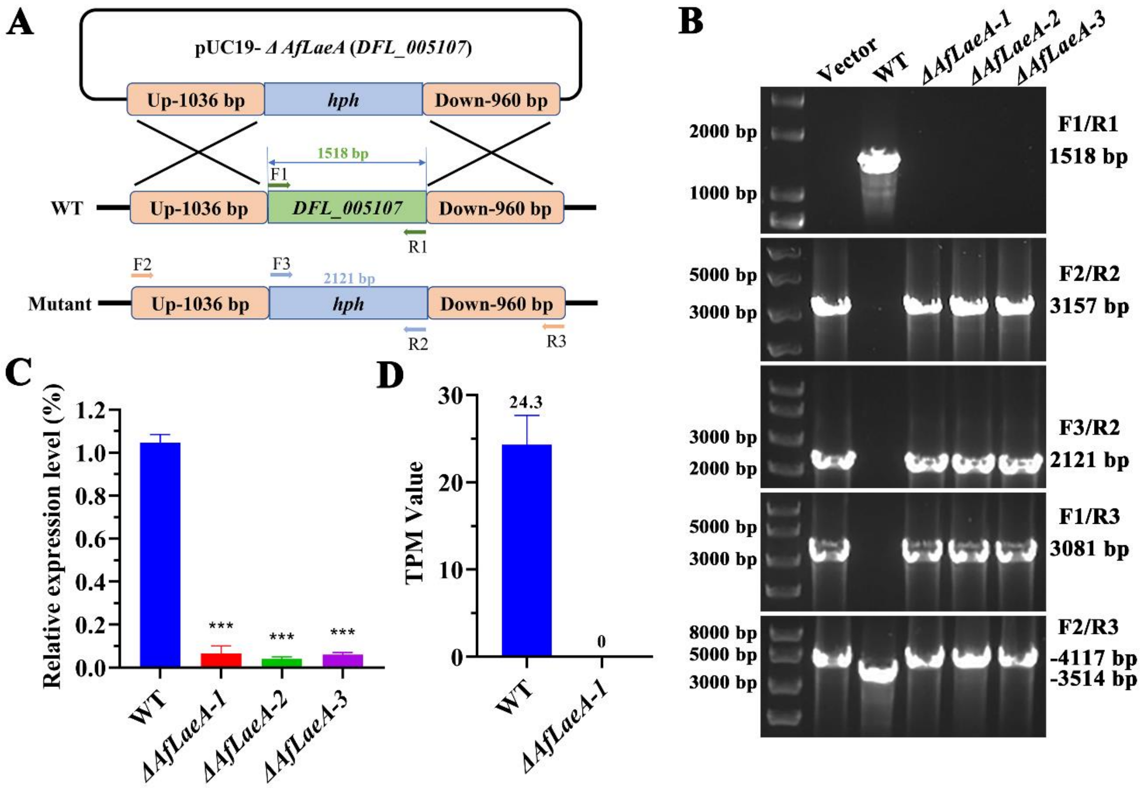

Figure S2 Disruption of *AfLaeA* gene in *A. flagrans.*

A, Diagram of the knockout pattern of the *AfLaeA* gene.

B, Detection of the knockout results of *AfLaeA* gene.

C, Detection of the relative expression levels of *AfLaeA* gene in *ΔAfLaeA* strains by qPCR.

D, Transcriptome datas were used to confirm the expression level *AfLaeA* gene in WT and *ΔAfLaeA* strains.

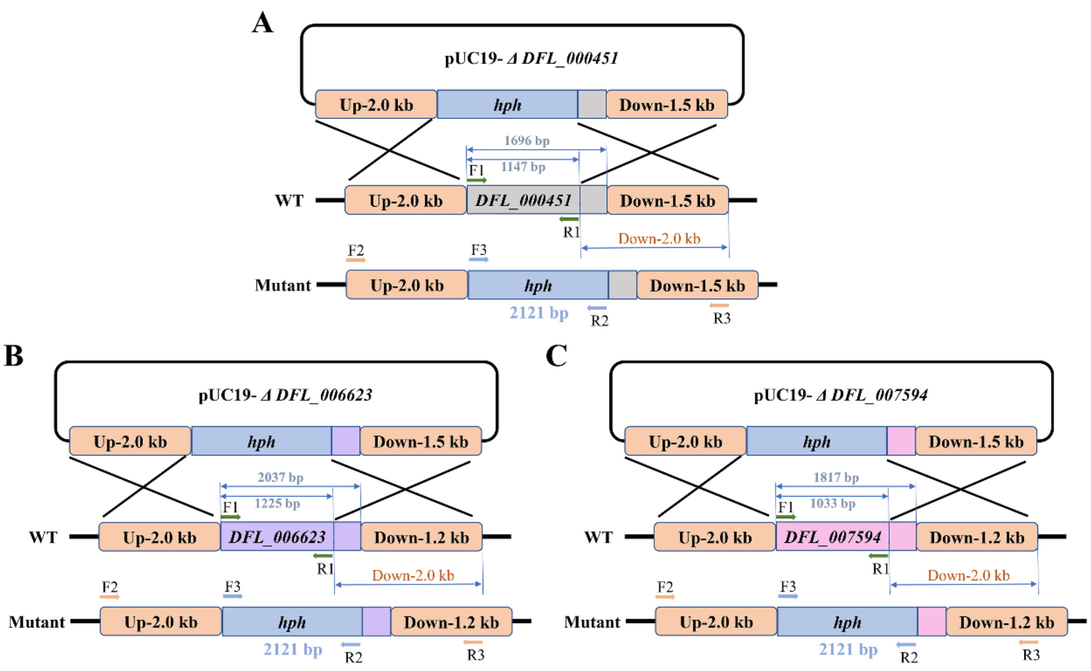

Figure S3 Disruption of three methyltransferases (*DFL_000451*, *DFL_006623*, and *DFL_007594*).

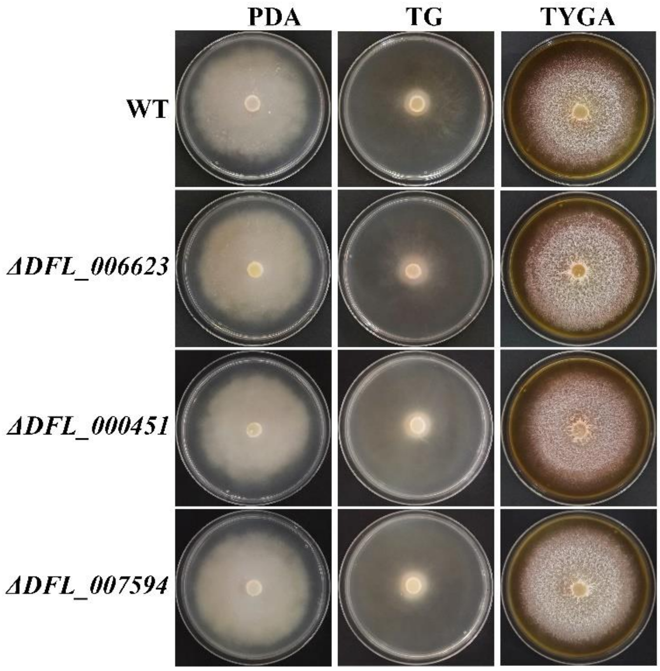

Figure S4 Analysis of the effects of three methyltransferase on mycelium growth. The knock-out strains of *DFL _ 000451*, *DFL _ 006623* and, *DFL _ 007594* genes were cultured with PDA, TG and, TYGA medium at 28℃ for 5 days, respectively.

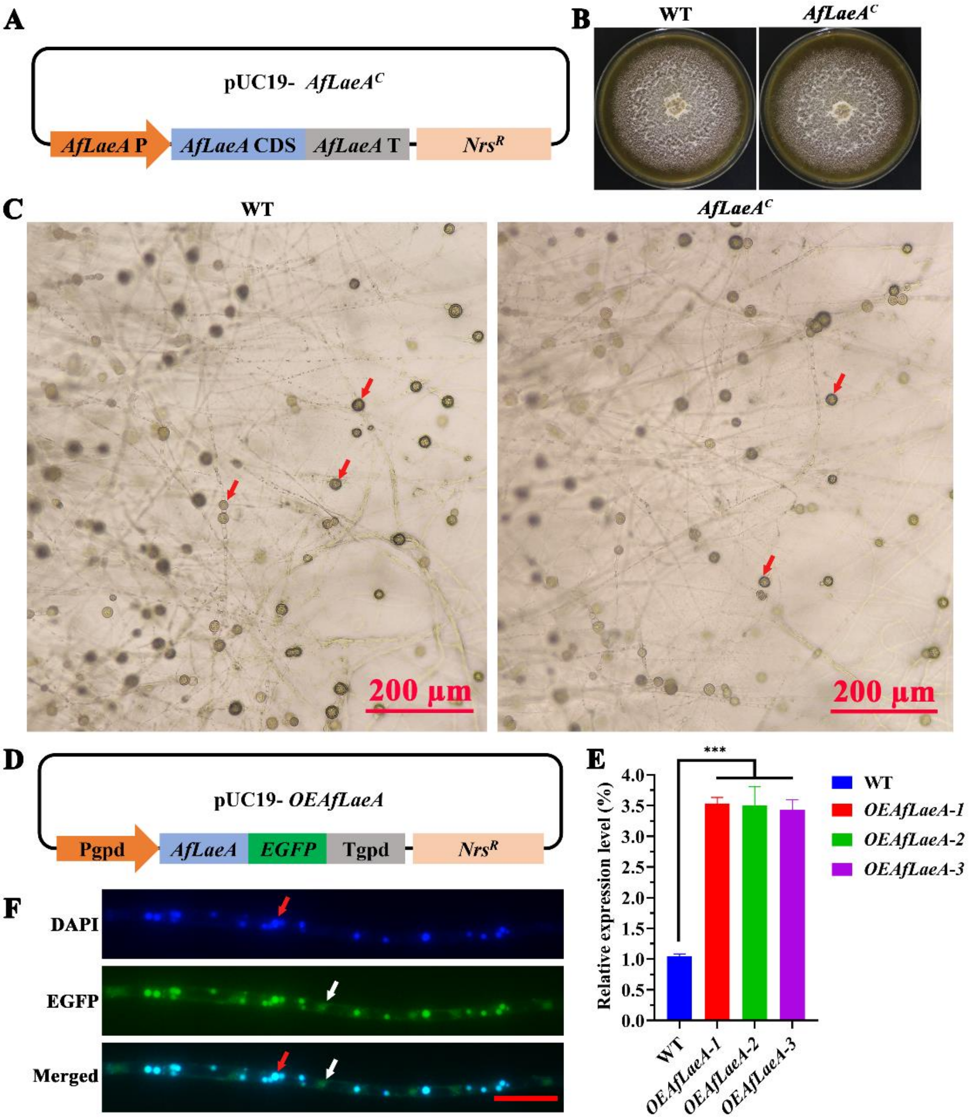

Figure S5 Complementation and overexpression of *AfLaeA* gene in the *ΔAfLaeA*, and WT strain, respectively.

A, Diagram of the complementation pattern of the *AfLaeA* gene by natural promoter in the *ΔAfLaeA* strain.

B, Growth of *A. flagrans* WT, and the complemented strains on TYGA after 7 days.

C, Overview of chlamydospores of the WT and the complemented strains. The red arrows indicate chlamydospores.

D, Diagram of the overexpression pattern of the *AfLaeA* gene.

E, Detection of the relative expression levels of *AfLaeA* gene in *OEAfLaeA* strains by qPCR.

F, Observtion of the localization of AfLaeA in cells by GFP fluorescence. Scale bars, 5 mm.

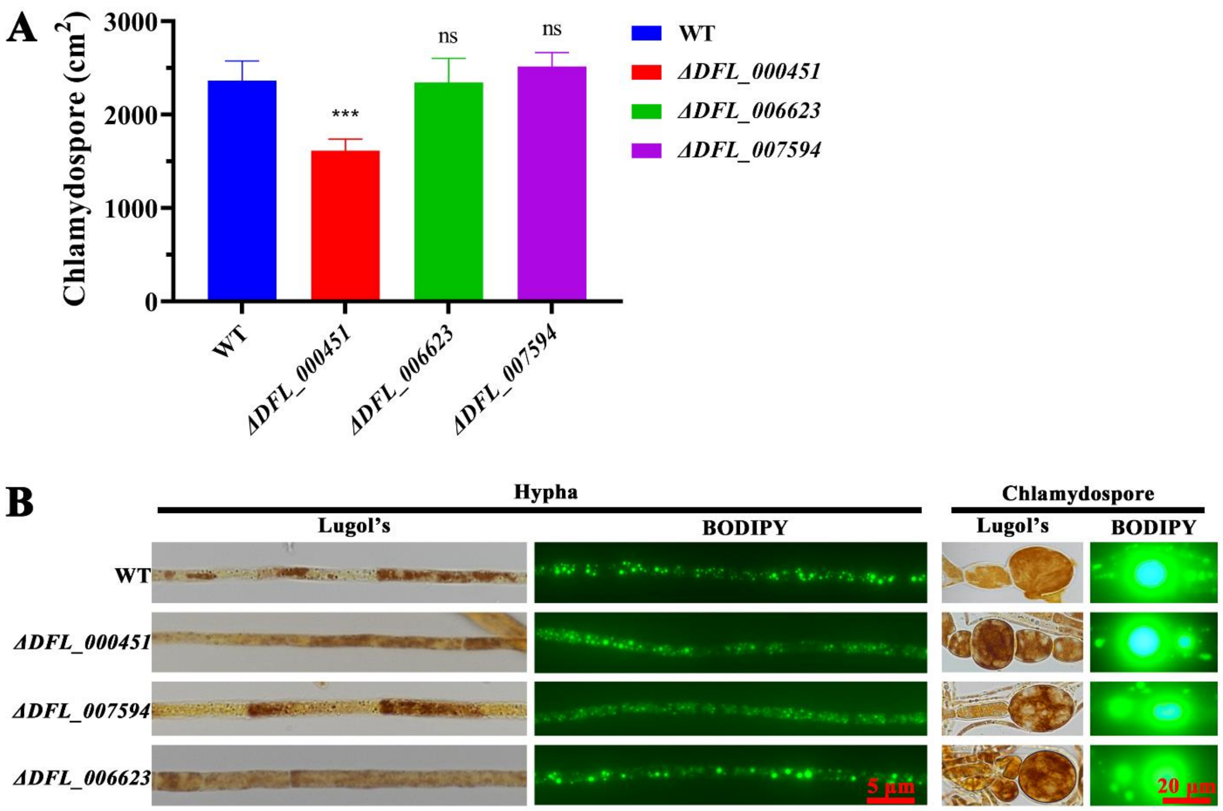

Figure S6 Analysis of the effects of *DFL_000451*, *DFL_006623* and *DFL_007594* genes on chlamydospore, lipid droplets and glycogen production.

A, Comparison of the number of chlamydospores between WT strain and *ΔDFL_000451*, *ΔDFL_006623*. and *ΔDFL_007594* strains (ns $P > 0.05$, *$P < 0.05$, **$P < 0.01$, ***$P < 0.001$).

B, Analysis of glycogen and LDs in *ΔDFL_000451*, *ΔDFL_006623*. and *ΔDFL_007594* strains.

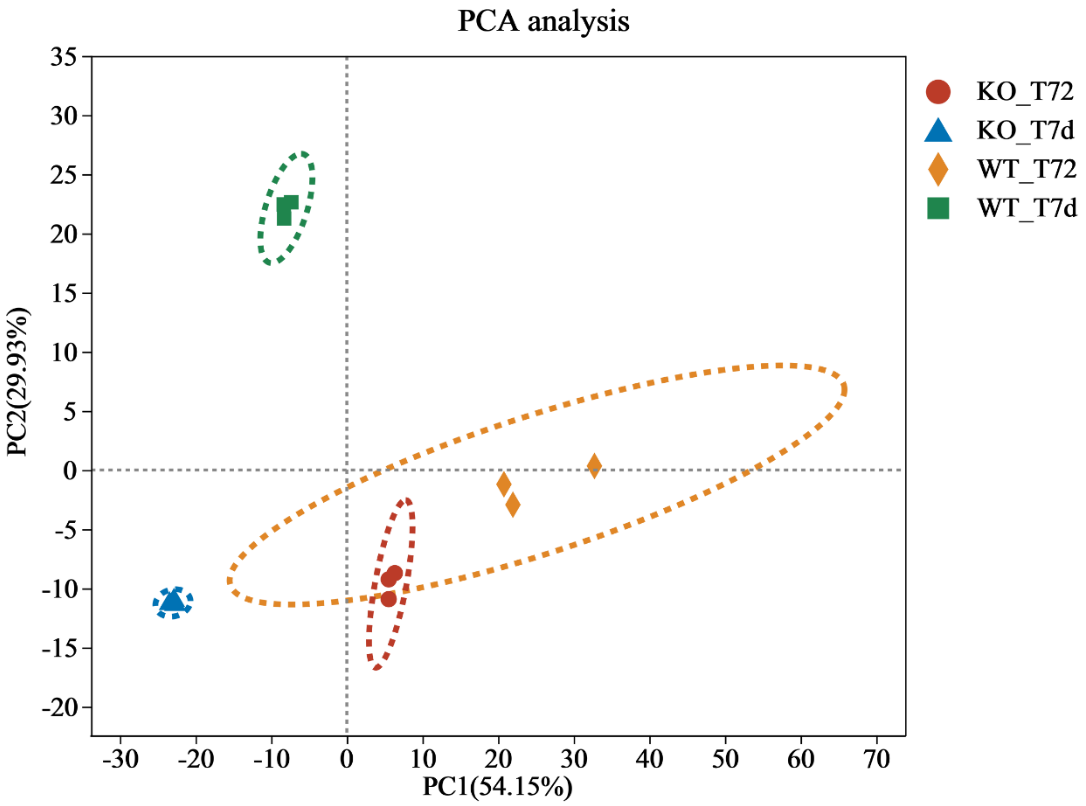

Figure S7 PCA analysis among samples. KO_T72 and WT_T72 represent the *AfLaeA* and WT strains were cultured for 3 days in TYGA medium, respectively. KO_T7d and WT_T7d represent the *ΔfLaeA* and WT strains were cultured for 7 days in TYGA medium, respectively.

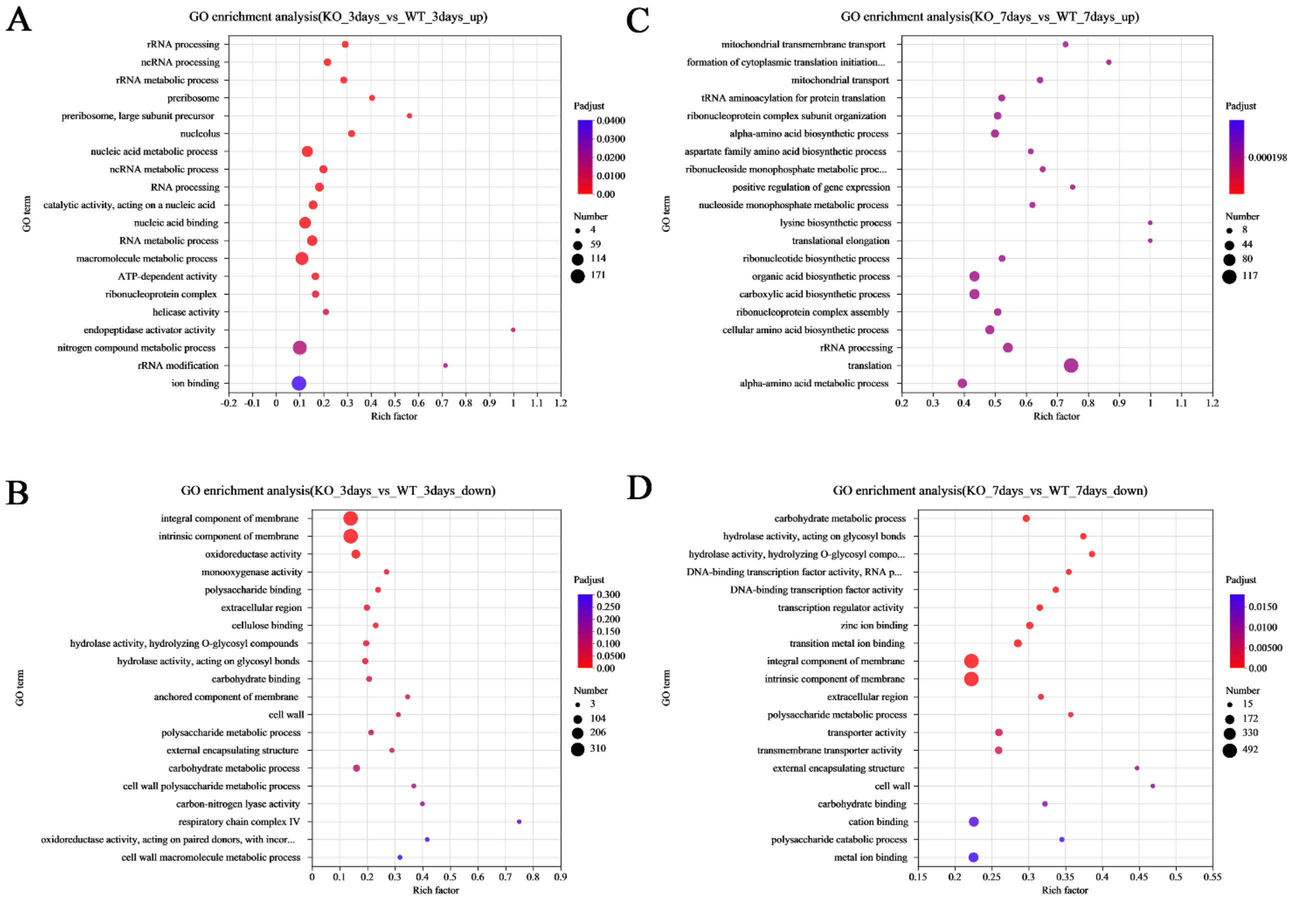

Figure S8 Gene Ontology (GO) enrichment analysis of the DEGs.

A and B, GO enrichment analysis of the upregulated and downregulated DEGs in the *ΔfLaeA* versus WT strain at 3 days.

C and D, GO enrichment analysis of the upregulated and downregulated DEGs in the *ΔfLaeA* versus WT strain at 7 days.

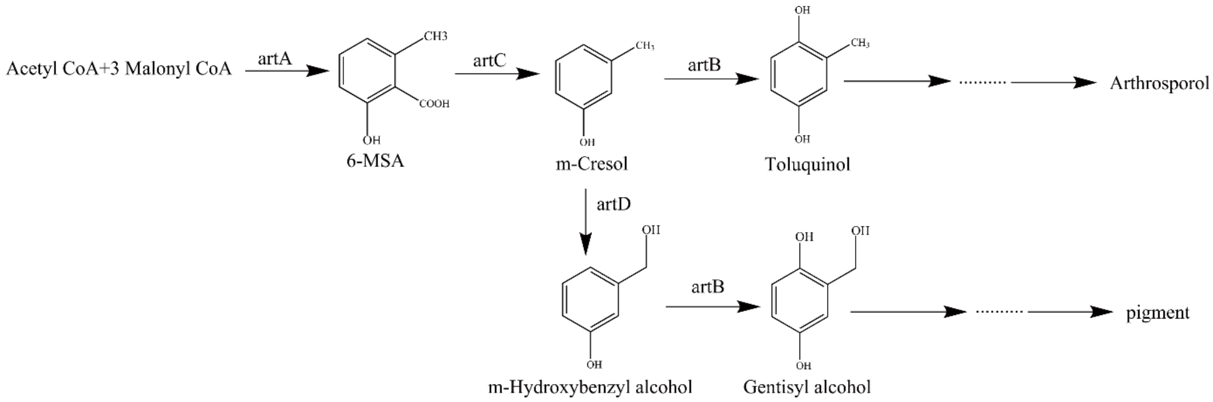

Figure S9 Proposed pathway for the biosynthesis of 6-MSA in *A. flagrans* and *A. oligospora* (1).

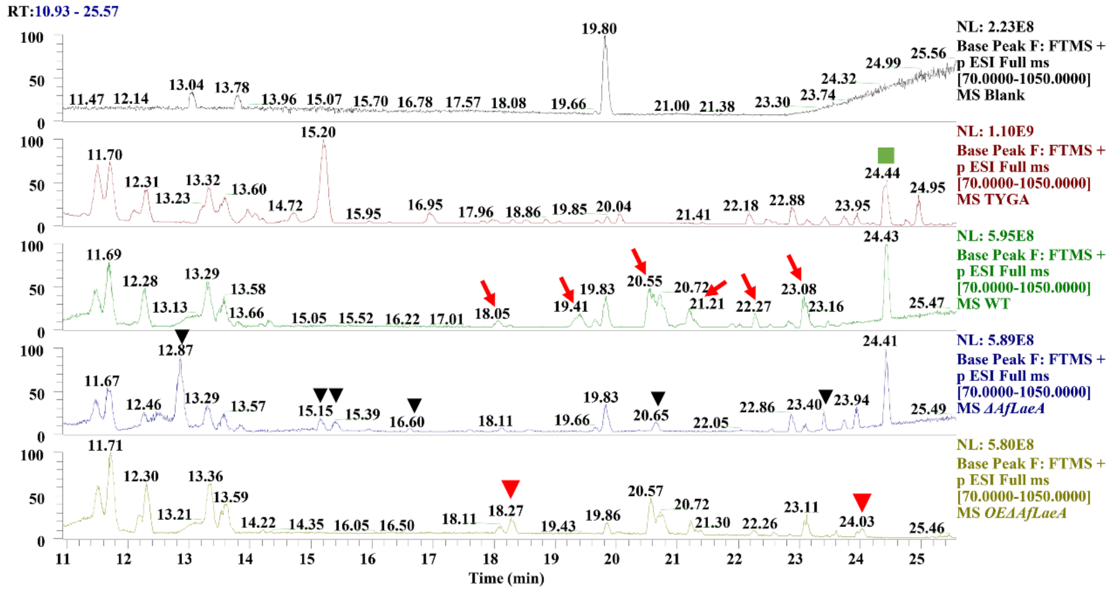

Figure S10 Comparative analysis of the metabolic profile of WT, *ΔAfLaeA*, and *OEAfLaeA* strains cultured in TYGA medium for 14 days by LC-MS. The red arrow indicated the missing components (at m/z 279.2309, 321.2392, 161.0596, and 303.2292 with retention times of 20.55, 21.21, 22.27, and 23.08 min, respectively) of *ΔAfLaeA* strain compared with WT strain. The black and red inverted triangles indicate the newly produced components (at m/z 130.0652, 511.2546, 130.0652, 494.2030, 279.2316, and 379.3354 with retention times of 12.87, 15.15, 15.39, 16.60, 20.65, and 23.40 min, respectively) of *ΔAfLaeA*, and *OEAfLaeA* strains, respectively. Green squares indicate the components with peak time of 24.44 min in TYGA medium, and cannot be used by WT and *ΔAfLaeA* strains.

Table S1 Analysis of the secondary metabolic gene clusters in *A. flagrans*.

#Gene_cluster	scaffold_id	Start	End	Length(bp)
r1c1	Lachesis_group0	1156002	1169634	13633
r1c2	Lachesis_group0	1950174	1959593	9420
r1c3	Lachesis_group0	5161964	5183546	21583
r2c1	Lachesis_group1	3721205	3772079	50875
r2c2	Lachesis_group1	4185164	4230526	45363
r3c1	Lachesis_group2	5483055	5504486	21432
r3c2	Lachesis_group2	5801941	5843543	41603
r4c1	Lachesis_group3	266356	314136	47781
r4c2	Lachesis_group3	724356	768791	44436
r4c3	Lachesis_group3	914956	947694	32739
r6c1	Lachesis_group5	399637	446941	47305

Table S2 Comparison of the conserved domain of methyltransferase between *A. nidulans* and *A. flagrans* by NCBI online tool Conserved Domain Search Service.

Protein					Domain Name	Description
AnLaeA (AAQ95166.1)	AfLaeA (DFL_005107) (EVM02G003030)	DFL_000451 (EVM05G004610)	DFL_006623 (EVM03G001000)	DFL_007594 (EVM03G010660)		
◆	●	▲	★	■	Methyltransf_25	Methyltransferase domain
◆	●	▲	★	■	Methyltransf_23	Methyltransferase domain
◆	●	▲	★	■	AdoMet_MTases	S-adenosylmethionine-dependent methyltransferases
◆	●	▲		■	Methyltransf_12	Methyltransferase domain
◆	●	▲	★	■	Methyltransf_11	Methyltransferase domain
	●	▲	★	■	PLN02336	phosphoethanolamine N-methyltransferase
◆	●	▲	★	■	HemK	Methylase of polypeptide chain release factors
◆	●	▲	★	■	Methyltransf_31	Methyltransferase domain
	●			■	Cfa	Cyclopropane fatty-acyl-phospholipid synthase and related methyltransferases
	●		★		PRK09328	N5-glutamine S-adenosyl-L-methionine-dependent methyltransferase
◆	●	▲	★	■	UbiE	Ubiquinone/menaquinone biosynthesis C-methylase
	●	▲	★	■	BioC	malonyl-acyl carrier protein O-methyltransferase
	●			■	PRK14968	putative methyltransferase
◆	●	▲	★	■	TrmN6	tRNA1(Val) A37 N6-methylase
	●	▲		■	ubiE	bifunctional demethylmenaquinone methyltransferase/2-methoxy-6-polyprenyl-1,4-benzoquinol methylase
	●		★	■	hemK_fam	HemK family putative methylases
	●	▲	★		MenG_MenH_UbiE	ubiquinone/menaquinone biosynthesis methyltransferases

	●				Methyltransf_16	Lysine methyltransferase
	●				PRK01544	bifunctional N5-glutamine S-adenosyl-L-methionine-dependent methyltransferase/tRNA (m7G46)
◆	●			■	MTS	Methyltransferase small domain
		▲	★	■	Tam	Trans-aconitate methyltransferase
		▲	★	■	Ubie_methyltran	ubiE/COQ5 methyltransferase family
		▲			PLN02244	tocopherol O-methyltransferase
		▲	★	■	SmtA	SAM-dependent methyltransferase
		▲			PTZ00098	phosphoethanolamine N-methyltransferase
◆		▲		■	PRK09328	N5-glutamine S-adenosyl-L-methionine-dependent methyltransferase
			★		PLN02490	MPBQ/MSBQ methyltransferase
			★		CMAS	Mycolic acid cyclopropane synthetase
			★	■	PRK07580	Mg-protoporphyrin IX methyl transferase
			★		PKS_MT	Methyltransferase in polyketide synthase (PKS) enzymes
			★		PRK01683	trans-aconitate 2-methyltransferase
				■	YrrM	Predicted O-methyltransferase YrrM
◆				■	RsmC	16S rRNA G1207 methylase RsmC
				■	PRK14103	trans-aconitate 2-methyltransferase
◆					FtsJ	FtsJ-like methyltransferase

Table S3 BLAST search of the hypothetical LaeA using AnLaeA (GeneBank: AAQ95166.1) as a query sequence in *A. flagrans* genome.

Gene ID	GeneBank	Percent Identity	Query Cover	E value
DFL_005107	RVD86853.1	48.47%	76%	6e-96
DFL_000451	RVD89443.1	37.15%	76%	1e-57
DFL_006623	RVD82190.1	37.92%	78%	5e-55
DFL_007594	RVD83196.1	35.26%	81%	2e-53
DFL_006144	RVD84392.1	31.76%	84%	2e-51
DFL_006658	RVD82225.1	34.00%	78%	4e-48
DFL_006575	RVD82141.1	33.77%	78%	1e-47
DFL_002107	RVD87904.1	31.80%	81%	4e-45
DFL_003019	RVD88847.1	27.57%	71%	7e-30
DFL_001934	RVD87721.1	25.29%	43%	3e-08

Table S4 The predicted and tallied physiochemical properties of AfLaeA, and three methyltransferase in *A. flagrans*.

Gene Name	Exon Number	Intron Number	Number of Amino Acids (aa)	Molecular Weight (kDa)	Subcellular localization	Reference
AnLaeA	-	-	374	43.0	Nuclear, Cytoplasmic	(2)
AfLaeA	7	6	362	41.491	Nuclear, Cytoplasmic	This study
DFL_000451	11	10	340	38.530	Cytoplasmic	This study
DFL_006623	12	11	367	40.620	Cytoplasmic	This study
DFL_007594	9	8	353	40.659	Cytoplasmic	This study

Table S5. Statistics of reads and mapping rate for the WT strain and *ΔAfLaeA* (KO) strains at different time points.

Sample	Total reads	Total mapped	Multiple mapped	Uniquely mapped
KO_T3d_3	43429426	41931260(96.55%)	718954(1.66%)	41212306(94.89%)
KO_T3d_2	42592746	40677347(95.5%)	788589(1.85%)	39888758(93.65%)
KO_T3d_1	50354928	48246991(95.81%)	905301(1.8%)	47341690(94.02%)
WT_T3d_3	48099230	45830147(95.28%)	1247476(2.59%)	44582671(92.69%)
WT_T3d_2	42921210	41080883(95.71%)	1016245(2.37%)	40064638(93.34%)
WT_T3d_1	56412094	53881198(95.51%)	1480983(2.63%)	52400215(92.89%)
KO_T7d_3	62833816	60393778(96.12%)	1243743(1.98%)	59150035(94.14%)
KO_T7d_2	60455376	58187374(96.25%)	1030325(1.7%)	57157049(94.54%)
KO_T7d_1	59156000	57426141(97.08%)	1080771(1.83%)	56345370(95.25%)
WT_T7d_3	64070156	61837821(96.52%)	1529355(2.39%)	60308466(94.13%)
WT_T7d_2	48599186	46912912(96.53%)	1095932(2.26%)	45816980(94.28%)
WT_T7d_1	50418794	48597415(96.39%)	1182796(2.35%)	47414619(94.04%)

Table S6 Relative content of unknown peaks in *ΔAfLaeA* and *ΔAfLaeA*^{RC} strains compared with WT (Top 20).

ΔAfLaeA					*ΔAfLaeA*^{RC}				
Predict molecular formula	Calc. MW	m/z	RT [min]	Ratio *ΔAfLaeA* /WT	Predict molecular formula	Calc. MW	m/z	RT [min]	Ratio *ΔAfLaeA*^{RC} /WT
$C_{20}H_{30}N_2O_2$	330.23056	331.23784	17.007	0.001771287	$C_{28}H_{36}N_4O$	444.2885	445.2958	17.186	1204.756
$C_{20}H_{32}N_2O$	316.25142	317.25869	16.951	0.002223715	$C_{28}H_{36}N_4O$	444.2887	445.2959	17.095	918.1334
$C_{10}H_{11}NOS$	193.05616	194.06344	16.929	0.003421061	$C_{20}H_{26}N_6O_4$	414.2022	415.2095	14.138	808.3236
$C_8H_{11}N_3O_3$	197.08009	198.08737	16.677	0.005762016	$C_{28}H_{36}N_4O$	444.2886	445.2959	15.75	588.2408
$C_{17}H_{14}O_7$	330.07386	331.08114	15.558	0.006608889	$C_9H_{23}N_4O_3P$	266.1498	267.157	12.46	558.5093
$C_{18}H_{15}N_3O$	289.12098	290.12826	16.977	0.007809357	$C_8H_{10}N_8O_2S$	282.0656	283.0728	0.522	549.7278
$C_{12}H_{27}NO$	201.20934	202.21662	15.651	0.008059129	$C_{20}H_{34}N_2O_3$	350.2568	351.264	11.036	487.2139
$C_{16}H_{10}O_6$	298.04755	299.05483	16.091	0.008960139	$C_{16}H_{27}N_4OP$	322.1939	323.2012	15.686	316.4279
$C_{16}H_{17}N_3$	251.14197	252.14925	15.65	0.009700595	$C_{14}H_{33}N_6O_2P$	348.2412	349.2484	11.677	316.3023
$C_{10}H_{11}NO_2S_2$	241.02307	242.03035	15.739	0.010699206	$C_4H_6N_2O$	98.04851	99.05572	13.616	314.4503
$C_{19}H_{30}N_2O$	302.2364	303.24368	16.944	0.01240193	$C_7H_6N_6OS$	222.0329	223.0402	10.416	294.5013
$C_{14}H_{32}N_6O_3S$	364.22448	365.2316	16.916	0.013988137	$C_{18}H_{33}N_4O_4P$	400.2231	401.2303	19.291	271.6765
$C_6H_5N_7O$	191.05591	192.06319	3.362	0.014213441	$C_{14}H_{33}N_6OP$	332.2461	333.2534	0.678	271.3978
$C_{28}H_{28}N_{10}O_{11}$	680.19516	681.20243	15.555	0.014471258	$C_{20}H_{34}N_2O_3$	350.2567	351.2639	0.85	269.5328
$C_9H_{21}N_4OP$	232.14446	233.15174	15.778	0.016945244	$C_6H_{19}N_7OS$	237.1364	238.1437	0.667	258.3884
$C_{20}H_{28}O_3$	316.2039	317.21128	18.564	0.017027088	$C_{11}H_{21}N_4O_2P$	272.1391	273.1464	17.081	255.7101
$C_{14}H_{31}NO_3$	261.2303	262.23757	16.101	0.017126961	$C_{11}H_{17}N_6O_2P$	296.116	297.1233	10.603	246.8178
$C_{20}H_{14}N_2O_2$	314.10519	315.11247	16.191	0.017209785	$C_4H_6N_2O$	98.04852	99.05572	13.841	245.8484545
$C_{11}H_{13}N_6OP$	276.0897	277.09698	16.83	0.018492191	$C_{20}H_{28}N_3P_3$	403.1507	404.1579	10.403	239.3218023
$C_{23}H_{35}N_3O_3$	401.26772	402.275	16.672	0.019293874	$C_{20}H_{34}N_2O_3$	350.2567	351.2641	0.638	221.8894215

Table S7 Presence and effects of *LaeA* in the fungal kingdom.

Fungal groups	Species	Function					Reference
		SM	G	D	V	SP	
Ascomycota	*Aspergillus nidulans*	SM	-	D	-	-	(3, 4)
	Aspergillus fumigatus	SM	-	D	V	-	(5, 6)
	Aspergillus oryzae	SM	-	D	-	-	(7, 8)
	Aspergillus flavus	SM	-	D	V	SP	(9, 10)
	Aspergillus carbonarius	SM	-	D	V	-	(11)
	Aspergillus luchuensis	SM	-	D	-	-	(12)
	Aspergillus niger	SM	G	D	-	-	(13, 14)
	Aspergillus terreus	SM	-	D	-	-	(15, 16)
	Aspergillus ochraceus	SM	-	D	V	-	(17)
	Aspergillus versicolor	SM	-	D	-	-	(18)
	Aspergillus pachycristatus	SM	-	D	-	-	(19)
	Aspergillus pseudoterreus	SM	-	D	-	-	(20)
	Aspergillus fumisynnematus	SM	-	D	-	-	(21)
	Monascus pilosus	SM	-	D	-	-	(22)
	Monascus ruber	SM	G	D	-	-	(23)
	Monascus purpureus	SM	-	D	-	-	(24)
	Trichoderma atroviride	SM	G	D	-	-	(25)
	Trichoderma reesei	SM	G	D		SP	(26, 27)
	Trichoderma afroharzianum	SM	-	-	-	-	(28)
	Trichoderma longibrachiatum SMF2	SM	G	D	-	SP	(29)
	Penicillium Brocae HDN-12-143	SM	-	-	-	-	(30)
	Penicillium dipodomyis YJ-11	SM	-	-	-	-	(31)
	Penicillium digitatum	SM	-	D	-	-	(32)
	Penicillium chrysogenum	SM	G	D	-	-	(33, 34)
	Penicillium citrinum	SM	-	D	-	-	(35, 36)
	Penicillium expansum	SM	G	D	V	-	(37, 38)
	Penicillium oxalicum	SM	-	D	-	-	(39)
	Fusarium verticillioides	SM	-	D	V	-	(40)
	Fusarium graminearum	SM	G	D	V	-	(41)
	Fusarium oxysporum	SM	G	D	V	-	(42)
	Fusarium fujikuroi	SM	-	D	V	-	(43)
	Cladosporium fulvum	SM	G	D	V	-	(44)
	Beauveria bassiana	SM	-	-	V	SP	(45)
	Cordyceps militaris	SM	-	-	-	-	(46)
	Botrytis cinerea	SM	-	D	V	SP	(47)
	Pestalotiopsis microspora	SM	G	D	V	-	(48)
	Valsa mali	SM	G	D	V	-	(49)
	Cochliobolus heterostrophus	SM	G	D	V	-	(50)
	Neurospora crassa	SM	G	D	V	-	(51)
	Chaetomium globosum	SM	G	D	V	-	(52)
	Dothistroma septosporum	SM	G	D	V	-	(53)
	Alternaria alternata	SM	G	D	V	-	(54, 55)
	Alternaria alstroemeria	SM	-	-	V	-	(56)
	Epichloe festucae	SM	G	D	V	SP	(57, 58)
	Magnaporthe oryzae	SM	-	D	V	-	(59)
	Pyricularia oryzae	SM	-	-	-	-	(60)
Basidiomycete	*Pleurotus ostreatus*	SM	G	D	-	SP	(61)
	Ganoderma lingzhi	SM	G	D	-	-	(62)
	Coprinopsis cinerea	SM	G	D	-	-	(63)

SM: secondary metabolite; G: Growth; D: fungal development; V: virulence; SP: secreted protein

Table S8 PCR primer sets used in this study.

Target gene	Sequence ID	Primers	Primer sequences (5'-3') [a]	Uses
AfLaeA	DFL_005107 (EVM02G003030)	Ko5107-up-for	ccgggtaccgagctcgaattcTTCGTTACCTTTTTTGCTTATTCCT	Amplify the 5’ flank of *AfLaeA* gene
		Ko5107-up-rev	cttctgTCAGACCAATTCCCCGGAA	
		Ko5107-hyg-for	gggaattggtctgaCAGAAGATGATATTGAAGGAGCATTT	Amplify the *hph* cassette
		Ko5107-hyg-rev	attgAAAGAAGGATTACCTCTAAACAAGTGTACC	
		Ko5107-down-for	gaggtaatccttctttCAATTATTAACAACTACGGCTCGC	Amplify the 3’ flank of *AfLaeA* gene
		Ko5107-down-rev	gtactgagagtgcaccatatgTTGGAAAGGTGGGGAATTAGG	
DFL_000451	DFL_000451 (EVM05G004610)	Ko0451-up-for	ccgggtaccgagctcgaattcCCCCAACGGTTCCTTTTGC	Amplify the 5’ flank of *DFL_000451* gene
		Ko0451-up-rev	catcttctgTGTGTCCAGTGCGGTTTGTCG	
		Ko0451-hyg-for	cactggacacaCAGAAGATGATATTGAAGGAGCATTT	Amplify the *hph* cassette
		Ko0451-hyg-rev	ccaggaggataAAAGAAGGATTACCTCTAAACAAGTGTACC	
		Ko0451-down-for	tccttctttTATCCTCCTGGTATCATTCAATATTAATAA	Amplify the 3’ flank of *DFL_000451* gene
		Ko0451-down-rev	gtactgagagtgcaccatatgTATGGTGCAAAGTAAATTGGTTTGA	
DFL_006623	DFL_006623 (EVM03G001000)	Ko6623-up-for	ccgggtaccgagctcgaattcTTTTGCGGTATATCTACAAATTCTAGTATAT	Amplify the 5’ flank of *DFL_006623* gene
		Ko6623-up-rev	tcatcttctgGGCTATAGACTTTTTTCTGGGGC	
		Ko6623-hyg-for	gtctatagccCAGAAGATGATATTGAAGGAGCATTT	Amplify the *hph* cassette
		Ko6623-hyg-rev	ccctatcatcggagaaaagtAAAGAAGGATTACCTCTAAACAAGTGTACC	
		Ko6623-down-for	ACTTTTCTCCGATGATAGGGTTCC	Amplify the 3’ flank of *DFL_006623* gene
		Ko6623-down-rev	gtactgagagtgcaccatatgCCAACTTCAAAGGAAAAGGATTAAA	
DFL_007594	DFL_007594 (EVM03G010660)	Ko7594-up-for	ccgggtaccgagctcgaattcGCTTCTTTTCGACTGTTGAAACC	Amplify the 5’ flank of *DFL_007594* gene
		Ko7594-up-rev	tctgCGATTTTAGAATAGGGAAAACCCT	
		Ko7594-hyg-for	ccctattctaaaatcgCAGAAGATGATATTGAAGGAGCATTT	Amplify the *hph* cassette
		Ko7594-hyg-rev	ggaaggataatggagagactAAAGAAGGATTACCTCTAAACAAGTGTACC	
		Ko7594-down-for	AGTCTCTCCATTATCCTTCCAAATCA	Amplify the 3’ flank of *DFL_007594* gene
		Ko7594-down-rev	gtactgagagtgcaccatatgGAAAATCGCACCGGGTAGTTG	
AfLaeA	DFL_005107 (EVM02G003030)	RC-5107-gpdp-for	ccgggtaccgagctcgaattcGATCACCTCGGCGGGGTC	Amplify the promoter of *Afgpd* gene
		RC-5107-gpdp-rev	ggtagacttctgtcatTTTGAATTATTGACTTTTGTCGAGG	
		RC-5107-cds-for	caaaATGACAGAAGTCTACCAATACGCTCA	Amplify the CDS fragment of *AfLaeA* gene
		RC-5107-cds-rev	ttgctcaccatAGGGCGTCGGGCAATCCA	
		RC-5107-egfp-for	cgacgccctATGGTGAGCAAGGGCGAGG	Amplify the *EGFP* fragment
		RC-5107-egfp-rev	gcttcacgctggggaattatTTACTTGTACAGCTCGTCCATGCC	
		RC-5107-gpdT-for	ATAATTCCCCAGCGTGAAGCT	Amplify the terminator of *Afgpd* gene
		RC-5107-gpdT-rev	gcctccatgtcATGTGGAAAAGGGATTTGATCG	
		RC-5107-nrsr-for	tttccacatGACATGGAGGCCCAGAATACC	Amplify the Nrs^R cassette

		RC-5107-nrsr-rev	gtactgagagtgcaccatatgCAGTATAGCGACCAGCATTCACA	
AfvosA	DFL_008845 (EVM00G008390)	AD-8390-for	gagtggccattatggATGCAAAATCATTCCTTTGCAGA	Amplify the CDS fragment of *AfveA* gene
		AD-8390-rev	gccgacatgttttttTCATGTAGCATAAGTTTGGCTAACAG	
AfveA	DFL_000190 (EVM05G002000)	AD-5070-for	gagtggccattatggATGGCCGACCGTTTTATCCC	Amplify the CDS fragment of *AfveA* gene
		AD-5070-rev	gccgacatgttttttTTACATGTCCATAGTATCACCCTGG	
AfvelB	DFL_008938 (EVM00G009330)	AD-9330-for	gagtggccattatggATGTATACTACAAGCTCTCGGGACC	Amplify the CDS fragment of *AfvelB* gene
		AD-9330-rev	gccgacatgttttttTCAGTAATCGTCATCATCGTCCTG	
AfvelC	DFL_004053 (EVM02G013760)	AD-3760-for	gagtggccattatggATGATGTCTCCTAGGCCTCCACC	Amplify the CDS fragment of *AfvelC* gene
		AD-3760-rev	gccgacatgttttttTTATGCTTTCCTGCGACGTTT	
AfpacC	DFL_007387 (EVM03G008580)	AD-8580-for	gagtggccattatggATGGATAGCACGGAACCACG	Amplify the CDS fragment of *AfpacC* gene
		AD-8580-rev	gccgacatgttttttTTATGCGGATGTTTCGACAGAC	
AfbrlA	DFL_001688 (EVM01G002650)	AD-2650-for	gagtggccattatggATGTCCCATCATAACATTCACATGG	Amplify the CDS fragment of *AfbrlA* gene
		AD-2650-rev	gccgacatgttttttTCAATGCTCAAGCTTGATCCC	
AfkapA	DFL_007032 (EVM03G005070)	AD-7032-for	catggaggccgaattcccgggATGGCCGACCGTTTTATCCC	Amplify the CDS fragment of *AfkapA* gene
		AD-7032-rev	gcaggtcgacggatccccgggTTACATGTCCATAGTATCACCCTGG	
AffphA	DFL_002824 (EVM01G014010)	AD-4010-for	gagtggccattatggATGGAAATCTTCAATATTTTGTCACAA	Amplify the CDS fragment of *AffphA* gene
		AD-4010-rev	gccgacatgttttttTCACCCTATGTGTGTCTGGAAGG	
AfLaeA	DFL_005107 (EVM02G003030)	AD-5107-for	catggaggccgaattcccgggATGACAGAAGTCTACCAATACGCTCA	Amplify the CDS fragment of *AfLaeA* gene
		AD-5107-rev	gcaggtcgacggatccccgggTTAAGGGCGTCGGGCAAT	
		BD-5107-for	catggaggccgaattcccgggATGACAGAAGTCTACCAATACGCTCA	
		BD-5107-rev	gcaggtcgacggatccccgggTTAAGGGCGTCGGGCAAT	
AfLaeA	DFL_005107 (EVM02G003030)	RC-5107-P-for	ccgggtaccgagctcgaattcGTTTCTATCATGAGCATGTATCCATG	Amplify the 5’ flank (2 kb) of *AfLaeA* gene
		RC-5107-P-rev	gtcatTCAGACCAATTCCCCGGAA	
		RC-5107-CDS-for	ggggaattggtctgaATGACAGAAGTCTACCAATACGCTCA	Amplify the CDS fragment of *AfLaeA* gene
		RC-5107-CDS-rev	taattgTTAAGGGCGTCGGGCAAT	
		RC-5107-T-for	cccgacgcccttaaCAATTATTAACAACTACGGCTCGC	Amplify the 3’ flank (1 kb) of *AfLaeA* gene
		RC-5107-T-rev	gcctccatgtcGAAGGAAAAGGGTGAAATTTGTCT	
		RC-5107-NRS-for	ttttccttcGACATGGAGGCCCAGAATACC	Amplify the NRS^R cassette
		RC-5107-NRS-rev	gtactgagagtgcaccatatgCAGTATAGCGACCAGCATTCACA	

[a]The primer sequence consistent with the vector is indicated by lowercase letters.

Table S9 Real time PCR primer sets used in this study.

Target gene	Sequence ID	Primers	Primer sequences (5'-3')
AfGpd	DFL_000198 (EVM05G002080)	*AfGpd*-for	CGAGAAGCCCGCCAAGT
		AfGpd-rev	AAAGGTGTCGGTCAAAGCAAT
AfLaeA	DFL_005107 (EVM02G003030)	RT-*AfLaeA*-for	TCTGGTAAACCACTTCGGTAC
		RT-*AfLaeA*-rev	GTGCGATGCAAAGGGAC
artA	DFL_002601 (EVM01G011780)	RT-*artA*-for	GCTCCCACATTTCCACCT
		RT-*artA*-rev	GGATTATCGGCATTCTGACTT
artB	DFL_002602 (EVM01G011790)	RT-*artB*-for	GCAGTTCAAACGCAGAGC
		RT-*artB*-rev	ATGGAAGAGCCGCAAAG
artC	DFL_002603 (EVM01G011800)	RT-*artC*-for	GTCCACGGAGGAAATAATCG
		RT-*artC*-rev	CTCGGAGAACTCGTACTTATCAA
artD	DFL_002604 (EVM01G011810)	RT-*artD*-for	TACTTGATGAGCCTCCACG
		RT-*artD*-rev	TTAGAAGACGCCCGATGA
artE	DFL_002606 (EVM01G011830)	RT-*artE*-for	TCACAAGATGGGCTCGGATTG
		RT-*artE*-rev	TAGCAGCGACGCCAGCAGT
artR	DFL_002600 (EVM01G011760)	RT-*artR*-for	CGAGACTTTGTGGGAGGA
		RT-*artR*-rev	ACTGTGGGACGTGATTGTG

Reference

1. Yu X, Hu X, Pop M, Wernet N, Kirschhöfer F, Brenner-Weiß G, Keller J, Bunzel M, Fischer R. 2021. Fatal attraction of *Caenorhabditis elegans* to predatory fungi through 6-methyl-salicylic acid. Nat Commun 12:5462.

2. Bayram O, Krappmann S, Ni M, Bok JW, Helmstaedt K, Valerius O, Braus-Stromeyer S, Kwon NJ, Keller NP, Yu JH, Braus GH. 2008. VelB/VeA/LaeA complex coordinates light signal with fungal development and secondary metabolism. Science 320:1504-1506.

3. Bok JW, Noordermeer D, Kale SP, Keller NP. 2006. Secondary metabolic gene cluster silencing in *Aspergillus nidulans*. Mol Microbiol 61:1636-1645.

4. Sarikaya Bayram O, Bayram O, Valerius O, Park HS, Irniger S, Gerke J, Ni M, Han KH, Yu JH, Braus GH. 2010. LaeA control of velvet family regulatory proteins for light-dependent development and fungal cell-type specificity. PLoS Genet 6:e1001226.

5. Ben-Ami R, Lewis RE, Leventakos K, Kontoyiannis DP. 2009. *Aspergillus fumigatus* inhibits angiogenesis through the production of gliotoxin and other secondary metabolites. Blood 114:5393-5399.

6. Lim FY, Hou Y, Chen Y, Oh JH, Lee I, Bugni TS, Keller NP. 2012. Genome-based cluster deletion reveals an endocrocin biosynthetic pathway in *Aspergillus fumigatus*. Appl Environ Microbiol 78:4117-4125.

7. Alshannaq AF, Gibbons JG, Lee MK, Han KH, Hong SB, Yu JH. 2018. Controlling aflatoxin contamination and propagation of *Aspergillus flavus* by a soy-fermenting *Aspergillus oryzae* strain. Sci Rep 8:16871.

8. Oda K, Kobayashi A, Ohashi S, Sano M. 2011. *Aspergillus oryzae laeA* regulates kojic acid synthesis genes. Biosci Biotechnol Biochem 75:1832-4.

9. Amaike S, Affeldt KJ, Yin WB, Franke S, Choithani A, Keller NP. 2013. The bZIP protein MeaB mediates virulence attributes in *Aspergillus flavus*. PLoS One 8:e74030.

10. Lv Y, Lv A, Zhai H, Zhang S, Li L, Cai J, Hu Y. 2018. Insight into the global regulation of laeA in *Aspergillus flavus* based on proteomic profiling. Int J Food Microbiol 284:11-21.

11. Maor U, Barda O, Sadhasivam S, Bi Y, Levin E, Zakin V, Prusky DB, Sionov E. 2021. Functional roles of LaeA, polyketide synthase, and glucose oxidase in the regulation of ochratoxin A biosynthesis and virulence in *Aspergillus carbonarius*. Mol Plant Pathol 22:117-129.

12. Kadooka C, Nakamura E, Mori K, Okutsu K, Yoshizaki Y, Takamine K, Goto M, Tamaki H, Futagami T. 2020. *LaeA* controls citric acid production through regulation of the citrate exporter-encoding *cexA* gene in *Aspergillus luchuensis* mut. *kawachii*. Appl Environ Microbiol 86: e01950-19.

13. Niu J, Arentshorst M, Nair PD, Dai Z, Baker SE, Frisvad JC, Nielsen KF, Punt PJ, Ram AF. 2015. Identification of a classical mutant in the industrial host *Aspergillus niger* by systems genetics: *LaeA* is required for citric acid production and regulates the formation of some secondary metabolites. G3 (Bethesda) 6:193-204.

14. Zhang M, Yang Y, Li L, Liu S, Xue X, Gao Q, Wang D, Zhang Y, Zhang J. 2022. *LaeA* regulates morphological development and ochratoxin A biosynthesis in *Aspergillus niger*. Mycotoxin Res 38:221-229.

15. Yao G, Chen X, Han Y, Zheng H, Wang Z, Chen J. 2022. Development of versatile and efficient genetic tools for the marine-derived fungus *Aspergillus terreus* RA2905. Curr Genet 68:153-164.

16. Wang B, Li X, Tabudravu J, Wang S, Deng H, Pan L. 2021. The chemical profile of activated secondary metabolites by overexpressing *LaeA* in *Aspergillus niger*. Microbiol Res 248:126735.

17. Wang G, Zhang H, Wang Y, Liu F, Li E, Ma J, Yang B, Zhang C, Li L, Liu Y. 2019. Requirement of LaeA, VeA, and VelB on asexual development, ochratoxin A biosynthesis, and fungal virulence in *Aspergillus ochraceus*. Front Microbiol 10:2759.

18. He T, Wang YD, Du LQ, Li FR, Hu QF, Cheng GG, Wang WG. 2020. Overexpression of global regulator *LaeA* induced secondary metabolite production in *Aspergillus versicolor* 0312. Rec Nat Prod 14:387-394.

19. Lan N, Yue Q, An Z, Bills GF. 2020. Apc.LaeA and Apc.VeA of the velvet complex govern secondary metabolism and morphological development in the echinocandin-producing fungus *Aspergillus pachycristatus*. J Ind Microbiol Biotechnol 47:155-168.

20. Pomraning KR, Dai Z, Munoz N, Kim YM, Gao Y, Deng S, Lemmon T, Swita MS, Zucker JD, Kim J, Mondo SJ, Panisko E, Burnet MC, Webb-Robertson BM, Hofstad B, Baker SE, Burnum-Johnson KE, Magnuson JK. 2022. Itaconic acid production is regulated by *LaeA* in *Aspergillus pseudoterreus*. Metab Eng Commun 15:e00203.

21. Hong EJ, Kim NK, Lee D, Kim WG, Lee I. 2015. Overexpression of the *laeA* gene leads to increased production of cyclopiazonic acid in *Aspergillus fumisynnematus*. Fungal Biol 119:973-983.

22 Lee SS, Lee JH, Lee I. 2013. Strain improvement by overexpression of the *laeA* gene in *Monascus pilosus* for the production of monascus-fermented rice. J Microbiol Biotechnol 23:959-965.

23. Liu QP, Cai L, Shao YC, Zhou YX, Li M, Wang XH, Chen FS. 2016. Inactivation of the global regulator LaeA in *Monascus ruber* results in a species-dependent response in sporulation and secondary metabolism. Fungal Biol 120:297-305.

24. Zhang C, Zhang H, Zhu Q, Hao S, Chai S, Li Y, Jiao Z, Shi J, Sun B, Wang C. 2020. Overexpression of global regulator *LaeA* increases secondary metabolite production in *Monascus purpureus*. Appl Microbiol Biotechnol 104:3049-3060.

25. Karimi Aghcheh R, Druzhinina IS, Kubicek CP. 2013. The putative protein methyltransferase LAE1 of *Trichoderma atroviride* is a key regulator of asexual development and mycoparasitism. PLoS One 8:e67144.

26. Karimi-Aghcheh R, Bok JW, Phatale PA, Smith KM, Baker SE, Lichius A, Omann M, Zeilinger S, Seiboth B, Rhee C, Keller NP, Freitag M, Kubicek CP. 2013. Functional analyses of *Trichoderma reesei* LAE1 reveal conserved and contrasting roles of this regulator. G3 (Bethesda) 3:369-378.

27. Fekete E, Karaffa L, Karimi Aghcheh R, Németh Z, Fekete E, Orosz A, Paholcsek M, Stágel A, Kubicek CP. 2014. The transcriptome of *lae1* mutants of *Trichoderma reesei* cultivated at constant growth rates reveals new targets of LAE1 function. BMC Genomics 15:447.

28. Ding Z, Wang X, Kong FD, Huang HM, Zhao YN, Liu M, Wang ZP, Han J. 2020. Overexpression of global regulator *Talae1* leads to the discovery of new antifungal polyketides from endophytic fungus *Trichoderma afroharzianum*. Front Microbiol 11:622785.

29. Shi JC, Shi WL, Zhou YR, Chen XL, Zhang YZ, Zhang X, Zhang WX, Song XY. 2020. The Putative methyltransferase TlLAE1 is involved in the regulation of peptaibols production in the biocontrol fungus *Trichoderma longibrachiatum* SMF2. Front Microbiol 11:1267.

30. Wang L, Zhang X, Zhang K, Zhang X, Zhu T, Che Q, Zhang G, Li D. 2020. Overexpression of global regulator *PbrlaeA* leads to the discovery of new polyketide in fungus *Penicillium Brocae* HDN-12-143. Front Chem 8:270.

31. Yu J, Han H, Zhang XY, Ma CT, Sun CX, Che Q, Gu QQ, Zhu TJ, Zhang GJ, Li DH. 2019. Discovery of two new sorbicillinoids by overexpression of the global regulator *LaeA* in a marine-derived fungus *Penicillium dipodomyis* YJ-11. Mar Drugs 17:446.

32. Zhu CY, Wang YY, Hu X, Lei MY, Wang MS, Zeng JW, Li HY, Liu ZY, Zhou T, Yu DL. 2020. Involvement of LaeA in the regulation of conidia production and stress responses in *Penicillium digitatum*. J Basic Microb 60:82-88.

33. Martín JF. 2017. Key role of LaeA and velvet complex proteins on expression of β-lactam and PR-toxin genes in *Penicillium chrysogenum*: cross-talk regulation of secondary metabolite pathways. J Ind Microbiol Biotechnol 44:525-535.

34. Vu TX, Vu HH, Nguyen GT, Vu HT, Mai LTD, Pham DN, Le DH, Nguyen HQ, Tran VT. 2019. A newly constructed Agrobacterium-mediated transformation system revealed the influence of nitrogen sources on the function of the LaeA regulator in *Penicillium chrysogenum*. Fungal Biol 123:830-842.

35. Xing W, Deng C, Hu C-H. 2010. Molecular cloning and characterization of the global regulator LaeA in *Penicillium citrinum*. Biotechnology Letters 32:1733-1737.

36. Baba S, Kinoshita H, Nihira T. 2012. Identification and characterization of *Penicillium citrinum* VeA and LaeA as global regulators for ML-236B production. Curr Genet 58:1-11.

37. Kumar D, Tannous J, Sionov E, Keller N, Prusky D. 2018. Apple intrinsic factors modulating the global regulator, LaeA, the patulin gene cluster and patulin accumulation during fruit colonization by *Penicillium expansum*. Front Plant Sci 9:1094.

38. Kumar D, Barad S, Chen Y, Luo XY, Tannous J, Dubey A, Matana NG, Tian SP, Li BQ, Keller N, Prusky D. 2017. LaeA regulation of secondary metabolism modulates virulence in *Penicillium expansum* and is mediated by sucrose. Mol Plant Pathol 18:1150-1163.

39. Zhang X, Zhu Y, Bao L, Gao L, Yao G, Li Y, Yang Z, Li Z, Zhong Y, Li F, Yin H, Qu Y, Qin Y. 2016. Putative methyltransferase LaeA and transcription factor CreA are necessary for proper asexual development and controlling secondary metabolic gene cluster expression. Fungal Genet Biol 94:32-46.

40. Butchko RAE, Brown DW, Busman M, Tudzynski B, Wiemann P. 2012. *Lae1* regulates expression of multiple secondary metabolite gene clusters in *Fusarium verticillioides*. Fungal Genetics and Biology 49:602-612.

41. Kim HK, Lee S, Jo SM, McCormick SP, Butchko RAE, Proctor RH, Yun SH. 2013. Functional roles of *FgLaeA* in controlling secondary metabolism, sexual development, and virulence in *Fusarium graminearum*. PLoS One 8:e68441.

42. Lopez-Berges MS, Schafer K, Hera C, Di Pietro A. 2014. Combinatorial function of velvet and AreA in transcriptional regulation of nitrate utilization and secondary metabolism. Fungal Genet Biol 62:78-84.

43. Wiemann P, Brown DW, Kleigrewe K, Bok JW, Keller NP, Humpf HU, Tudzynski B. 2010. FfVel1 and FfLae1, components of a velvet-like complex in *Fusarium fujikuroi*, affect differentiation, secondary metabolism and virulence. Mol Microbiol 77:972-994.

44. Griffiths S, Saccomanno B, de Wit P, Collemare J. 2015. Regulation of secondary metabolite production in the fungal tomato pathogen *Cladosporium fulvum*. Fungal Genet Biol 84:52-61.

45. Qin YQ, Ortiz-Urquiza A, Keyhani NO. 2014. A putative methyltransferase, mtrA, contributes to development, spore viability, protein secretion and virulence in the entomopathogenic fungus *Beauveria bassiana*. Microbiol 160:2526-2537.

46. Rachmawati R, Kinoshita H, Nihira T. 2018. Production of insect toxin beauvericin from entomopathogenic fungi *Cordyceps militaris* by heterologous expression of global regulator. Agrivita 40:177-184.

47. Wei Z, Shu D, Sun Q, Chen DB, Li ZM, Luo D, Yang J, Tan H. 2022. The BcLAE1 is involved in the regulation of ABA biosynthesis in *Botrytis cinerea* TB-31. Front Microbiol 13:969499.

48. Akhberdi O, Zhang Q, Wang D, Wang HC, Hao XR, Liu YJ, Wei DS, Zhu XD. 2018. Distinct roles of Velvet complex in the development, stress tolerance, and secondary metabolism in *Pestalotiopsis microspora*, a Taxol Producer. GENES 9:164.

49. Feng YQ, Yin ZY, Wu YX, Xu LS, Du HX, Wang NN, Huang LL. 2020. LaeA controls virulence and secondary metabolism in apple canker pathogen *Valsa mali*. Front Microbiol 11:581203.

50. Wu DL, Oide S, Zhang N, Choi MY, Turgeon BG. 2012. ChLae1 and ChVel1 Regulate T-toxin production, virulence, oxidative stress response, and development of the maize pathogen *Cochliobolus heterostrophus*. Plos Pathog 8:e1002542.

51. Bayram OS, Dettmann A, Karahoda B, Moloney NM, Ormsby T, McGowan J, Cea-Sanchez S, Miralles-Duran A, Brancini GTP, Luque EM, Fitzpatrick DA, Canovas D, Corrochano LM, Doyle S, Selker EU, Seiler S, Bayram O. 2019. Control of development, secondary metabolism and light-dependent carotenoid biosynthesis by the Velvet complex of *Neurospora crassa*. Genetics 212:691-710.

52. Cheng M, Zhao SS, Lin CY, Song JZ, Yang Q. 2021. Requirement of LaeA for sporulation, pigmentation and secondary metabolism in *Chaetomium globosum*. Fungal Biol 125:305-315.

53. Chettri P, Bradshaw RE. 2016. LaeA negatively regulates dothistromin production in the pine needle pathogen *Dothistroma septosporum*. Fungal Genet Biol 97:24-32.

54. Estiarte N, Lawrence CB, Sanchis V, Ramos AJ, Crespo-Sempere A. 2016. LaeA and VeA are involved in growth morphology, asexual development, and mycotoxin production in *Alternaria alternata*. Int J Food Microbiol 238:153-164.

55. Takao K, Akagi Y, Tsuge T, Harimoto Y, Yamamoto M, Kodama M. 2016. The global regulator LaeA controls biosynthesis of host-specific toxins, pathogenicity and development of *Alternaria alternata* pathotypes. J Gen Plant Pathol 82:121-131.

56. Yin X, Han L, Zheng W, Cai L, Qin M, He Z, Kang J. 2022. Global regulatory factor AaLaeA upregulates the production of antitumor substances in the endophytic fungus *Alternaria alstroemeria*. J Basic Microbiol 62:1402-1414.

57. Rahnama M, Maclean P, Fleetwood DJ, Johnson RD. 2020. VelA and LaeA are key regulators of *Epichloe festucae* transcriptomic response during symbiosis with perennial ryegrass. Microorganisms 8:33.

58. Rahnama M, Maclean P, Fleetwood DJ, Johnson RD. 2019. The LaeA orthologue in *Epichloë festucae* is required for symbiotic interaction with Lolium perenne. Fungal Genet Biol 129:74-85.

59. Saha P, Ghosh S, Roy-Barman S. 2020. *MoLAEA* regulates secondary metabolism in *Magnaporthe oryzae*. mSphere 5: e00936-19.

60. Yun CS, Motoyama T, Osada H. 2017. Regulatory mechanism of mycotoxin tenuazonic acid production in *Pyricularia oryzae*. ACS Chem Biol 12:2270-2274.

61. Zhang G, Yan P, Leng DD, Shang L, Zhang CH, Wu ZW, Wang ZH. 2022. Functional roles of *LaeA*-like genes in fungal growth, cellulase activity, and secondary metabolism in *Pleurotus ostreatus*. J Fungi 8:902.

62. Luo Q, Li N, Xu JW. 2022. A methyltransferase LaeA regulates ganoderic acid biosynthesis in *Ganoderma lingzhi*. Front Microbiol 13: 1025983.

63. Tsunematsu Y, Takanishi J, Asai S, Masuya T, Nakazawa T, Watanabe K. 2019. Genomic mushroom hunting decrypts coprinoferrin, a siderophore secondary metabolite vital to fungal cell development. Org Lett 21:7582-7586.
